# Systematic genetic assessment of hearing loss using whole-genome sequencing identifies pathogenic variants

**DOI:** 10.1038/s12276-025-01428-x

**Published:** 2025-04-01

**Authors:** Jung Ah Kim, Seung Hyun Jang, Sun Yung Joo, Se Jin Kim, Jae Young Choi, Jinsei Jung, Heon Yung Gee

**Affiliations:** 1 Department of Pharmacology, Graduate School of Medical Science, Brain Korea 21 Project, Yonsei University College of Medicine, Seoul, Republic of Korea; 2 Won-Sang Lee Institute for Hearing Loss, Seoul, Republic of Korea; 3 Department of Otorhinolaryngology, Yonsei University College of Medicine, Seoul, Republic of Korea

**Keywords:** Peripheral neuropathies, Disease genetics

## Abstract

Hearing loss is a clinically and genetically heterogeneous sensorineural disease that affects approximately 1 out of 1000 newborns. For the molecular diagnosis of genetic hearing loss, target panel or whole-exome sequencing (WES) have been widely used due to their cost-effectiveness and efficacy. Despite the advantages of WES, the plausible diagnoses in a substantial number of patients remain elusive due to its limited coverage. Here we utilized whole-genome sequencing (WGS) on a large cohort of individuals with hearing loss to overcome the drawbacks of WES and find the advantages of WGS. We implemented a systematic workflow to identify coding region variants, cryptic splice variants, mitochondrial variants, copy number variants, *cis*-regulatory variants and transposable element insertions. WGS was conducted on 140 families with hearing loss. Causative variations were identified in 37 of these families, accounting for 26% of the total. WGS possessed the capability to find genetic variations that are not identifiable using WES. The identified variants by WGS in this study encompassed aberrant splicing variants in *EYA1* and *CDH23*, mitochondrial variants in *MT-RNR1* and *MT-CO1*, structural variants in *STRC*, and Alu insertion in *SLC17A8*. These findings highlight the benefits of WGS. With the decreasing cost of WGS, its usage will become more prevalent, allowing more precise identification of the genetic causes of hearing loss.

## Advanced hearing loss diagnosis with whole-genome sequencing

Hearing loss is a common condition that affects many people worldwide. Understanding its genetic causes can help physicians provide better treatments. Researchers have used whole-exome sequencing (WES) to study these genes, but this method covers only a small part of the genome. This study explores the use of whole-genome sequencing (WGS), which examines the entire genome, to find more genetic causes of hearing loss. The study involved 577 families with hearing loss. Researchers performed WGS on 140 families and compared these results with those obtained from performing WES on 437 families. They looked for genetic variants in known hearing loss genes and other parts of the genome. WGS was able to identify additional variants that WES missed, such as those in noncoding regions and mitochondrial DNA. The results showed that WGS could find more potential causes of hearing loss than WES.

This summary was initially drafted using artificial intelligence, then revised and fact-checked by the author.

## Introduction

Hearing loss is the most common sensory disorder, affecting 1.33 per 1,000 newborns in developed countries^1^ and about half of individuals aged over 60 years (ref.^2^). It has a substantial impact on an individual’s quality of life. Accurate molecular diagnosis is crucial for identiyfing the genetic causes of hearing loss, allowing clinicians to forecast patient outcomes and select suitable therapeutic interventions^3^. However, achieving accurate genetic diagnoses in patients with hearing loss can be challenging due to the genetic heterogeneity and variable phenotypic expressivity of the condition^4^.

Whole-exome sequencing (WES) has been widely used for genetic diagnosis in patients with hearing loss. WES primarily targets the exome, which represents only a small portion, approximately 1–2%, of the entire genome. Regarding hearing loss, numerous efforts for deciphering genetic etiologies underlying hearing loss have been made in different ethnicities by WES and targeted exome sequencing. For example, Guan et al. developed a tiered exome-sequencing-based comprehensive gene panel, named AUDIOME, for the diagnosis of heterogeneous nonsyndromic sensorineural hearing loss (SNHL), and screening resulted in a diagnostic yield of 33.3% (11/33)^5^. Shearer et al. developed a platform, OtoSCOPE, that couples targeted genomic enrichment and massively parallel sequencing to capture and sequence exon regions of genes associated with nonsyndromic hearing loss, and the overall diagnostic rate was 42% (42/100)^6^. In Australia, 56% (59/106) of the molecular causes of congenital hearing impairment in a population-derived 2-year birth cohort of infants were diagnosed by WES^7^. Besides, targeted genome sequencing of 1,027 Chinese patients with bilateral hearing loss revealed 57.3% (558/1027) of genetic causes^8^, and clinical exome sequencing of 47 English patients with SNHL resulted in diagnostic yield of 38.3% (18/47)^9^. In France, a combined multistep strategy including DFNB1 locus analysis, targeted massive parallel exome sequencing of 74 genes, and additional approaches including copy number variation, in silico analyses and minigene studies was conducted among French families with deafness, and causative variants were discovered in 47.8% of families (99/207)^10^. In the Republic of Korea, many studies reported the identification of pathogenic variants by WES in patients with deafness, and functional validation of those variants was performed^11–16^.

Even though WES and clinical panel screening substantially contributed to the diagnosis of genetic hearing loss and has provided valuable insights into the genetic basis of hearing loss, WES techniques still have many limitations. For instance, WES cannot include regulatory or intronic regions that are known to be associated with a specific phenotype^17^. Furthermore, the ability to detect copy number variants (CNVs), especially those affecting one or a few exons, is so limited that important structural variants may be overlooked^17^. When multiple types of exome are available, WES is limited by the fact that not all tests are equivalent^17^.

In recent years, whole-genome sequencing (WGS) has emerged as a powerful tool for genetic analysis. Indeed, WGS is considered an initial test for suspected mendelian conditions and also appropriate when no candidate variants are detected by WES^17^. In contrast to WES, WGS covers the entire genome, offering a more thorough perspective of the genetic landscape. This broader coverage of the genome enables the detection of variants in *cis*-regulatory regions, deep-intronic splicing alterations, mitochondrial DNA (mtDNA) variants and structural variants, which are increasingly recognized as critical contributors to gene expression and disease pathogenesis^18–20^.

In this study, we sought to evaluate the diagnostic capabilities of WGS for patients with hearing loss. We hypothesized that WGS would provide additional insights into the genetic etiology of hearing loss by uncovering putative disease-causing variants that are difficult to be detected by WES. Here, we present the diagnostic yield of WGS and discuss the clinical utility of WGS in improving molecular diagnoses, predicting patient prognosis and guiding personalized treatment strategies.

## Materials and methods

### Study design and patients

This study received approval from the institutional review board of Severance Hospital, Yonsei University Health System (IRB #4-2015-0659). Individuals with hearing loss were enrolled in the Yonsei University Hearing Loss (YUHL) cohort, and they provided informed consent for the participation in the study and publication of their clinical data. Among the total 729 enrolled families, WES was performed on probands from 437 families, while probands from 140 families underwent WGS.

### Clinical evaluation

All enrolled patients underwent a comprehensive physical examination and history interviews. Audiological evaluations were conducted for all patients, as well as their affected and unaffected family members, using appropriate tests such as pure-tone audiometry, otoacoustic emission test, auditory brain stem response test or auditory steady-state response test. For pure-tone audiometry, air and bone conduction thresholds were measured in a double-walled audio booth, with frequencies ranging from 250 to 8000 Hz for air conduction and 250 to 4000 Hz for bone conduction. The degree of hearing loss was categorized as mild (26–40 dB), moderate (41–70 dB), severe (71–90 dB) or profound (>90 dB), based on the average threshold of the four frequencies (500, 1000, 2000 and 4000 Hz). The audiogram pattern was defined as ascending when the average thresholds for high frequencies (2000 Hz and 4,000 Hz) were at least 25 dB lower than those for low frequencies (250 and 500 Hz), ski-sloping when high frequencies were more than 25 dB higher than low frequencies, and flat when the difference between high and low frequencies was within 25 dB.

### Variant calling

Genomic DNA was extracted from peripheral blood obtained from the affected individuals and their parents (whenever available) using red blood cell lysis, cell lysis and protein precipitation solutions (iNtRon Biotechnology). The integrity of the genomic DNA was checked by running an agarose gel electrophoresis, and genomic DNA was quantified using fluorometry (Qubit, Invitrogen). The sequencing libraries were prepared according to the manufacturer’s instructions of TruSeq DNA Nano Library Prep Kit. (Illumina). In brief, 100 ng of genomic DNA from each sample were fragmented by acoustic shearing on a Qsonica 800 R2 instrument. Fragments of 350 bp were ligated to Illumina’s adapters and polymerase chain reaction (PCR)-amplified. An appropriate size for the final library is 500–600 bp. Libraries were quantified using the TapeStation 4200 instrument (Agilent Technologies) and KAPA Library Quantification Kit (KK4824, Kapa Biosystems). The resulting purified libraries were applied to an Illumina flow cell for cluster generation and sequenced using 150-bp paired-end reads on an Illumina NovaSeq 6000 (Illumina) sequencer by following the manufacturer’s protocols. The average sequencing depth of the libraries was 30×. Genomic Analysis Toolkit (GATK) best-practice pipelines were used to generate a binary alignment map (BAM) and variant call format (VCF) files from raw unmapped reads. The human reference genome GRCh38/hg38 was used to align the reads using the Burrows–Wheeler Aligner (BWA-MEM) algorithm. HaplotypeCaller was used to generate genotype VCF (gVCF) files for each sample. To filter out low-quality single-nucleotide variants (SNVs), a filter was applied with a coverage depth (CD) ≥5 and genotype quality (GQ) ≥20. To screen cytomegalovirus (CMV) infection among WGS samples, reads that were not mapped to the human reference genome were extracted using samtools (v1.3.0). Later, these unmapped reads were converted to the raw sequencing reads using bamToFastq of bedtools (v2.30.0), and paired fastq was realigned to the CMV reference genome (NCBI Accession NC_006273.2) with the BWA-MEM algorithm. The number and proportion of mapped reads to viral genome were calculated using flagstat function of samtools (v1.3.0). For mitochondrial short-variant discovery, GATK best-practice pipelines were used. Unlike germline-derived variants, mitochondrial variants were called using Mutect2. Blacklisted regions in mitochondrial genome were filtered, and variants having a ‘PASS’ flag in the ‘FILTER’ field were used for downstream analysis to remove false-positive calls. For CNV calling, a read-depth-based algorithm, CNVnator (v0.3.2, https://github.com/abyzovlab/CNVnator), was used as previously reported. For calling of transposable element (TE) insertion (TEI) from WGS data, computational tool xTea (v0.1.9, https://github.com/parklab/xTea) was used as reported.

### Coverage calculation

Coverage was calculated on 19 probands who had undergone both WES and WGS for precise comparison. The depth of sequencing coverage was calculated using the tool Mosdepth (v0.3.3, https://github.com/brentp/mosdepth). For WES samples, an Agilent SureSelect V5 enrichment capture bed was given as interval options and the depth of corresponding regions was calculated. Gene coverage was calculated using DepthOfCoverage from GATK. First, among known hearing loss genes, only regions overlapping with an Agilent SureSelect V5 enrichment capture were obtained by bedtools (v2.30.0, https://github.com/arq5x/bedtools). We divided the percentage of a given region covered above 15 into 10 bins and counted the number of intervals in each sample that corresponded to each percentage bin. We then averaged the number of those intervals across samples, converted them to percentages and plotted them as a pie chart. Three main quality parameters, CD, GQ and minor read ratio (MRR) were calculated as previously reported^21^ and source code used for this analysis is publicly available via GitHub (https://github.com/HGID/WES_vs_WGS).

### Annotation and classification of candidate variants in known NSHL genes

The SNVs and small indels were annotated using ANNOVAR software. To identify rare variants of unknown significance, variants were filtered using total minor allele frequency (MAF) in the Genome Aggregation Database (gnomAD) with a cutoff of 0.005. Ethnicity-specific MAFs and POPMAX 95% confidence interval estimates were compared within gnomAD. Novel variants that are not reported in the genome aggregation database were also selected. The variants were prioritized using various in silico prediction scores. The pathogenicity of missense variants was predicted using prediction scores from at least five prediction tools: SIFT (https://sift.bii.a-star.edu.sg/), PolyPhen2 (http://genetics.bwh.harvard.edu/pph2/), MutationTaster2 (http://www.mutationtaster.org/), CADD (https://cadd.gs.washington.edu/) and REVEL (https://sites.google.com/site/revelgenomics/). The interpretation of variants was based on the clinical interpretation of the ClinVar (https://www.ncbi.nlm.nih.gov/clinvar/), Human Gene Mutation Database (http://www.hgmd.cf.ac.uk/ac/index.php) and Deafness Variation Database (https://deafnessvariationdatabase.org/) to identify previously reported pathogenic or likely pathogenic variants. If no interpretation was registered, the identified variants were classified as variants of unknown significance. Cryptic splice site variants were identified using SpliceAI as a plugin via Ensembl’s Variant Effect Predictor tool (v106, https://github.com/Ensembl/ensembl-vep) and filtered by a delta score threshold of 0.1 in transcribed regions of genes. For CNV, identified regions were annotated using a AnnotSV (v2.2, https://github.com/lgmgeo/AnnotSV). To increase confidence, only CNV regions >1 kb in size and variants with a ranking score above 3 from AnnotSV were selected. All candidate CNVs were manually confirmed via genome-wide visualization against control samples utilizing Integrative genomics viewer (IGV) (v2.13.2, https://github.com/igvteam/igv). Mitochondrial variants were additionally annotated using MITOMAP database (https://www.mitomap.org/MITOMAP) and prioritized by a disease status of ‘Cfrm’ or ‘Reported’ for deafness. Mitochondrial variants having GenBank or gnomAD MAF under 0.005 and representing maternal inheritance by segregation analysis were selected as causative variants. Among causative variants, variants that were previously reported as pathogenic when considering the ethnicity were regarded as highly pathogenic variants. As for TEIs, only insertions predicted by xTea as ‘two-sided target-primed reverse transcription’ were considered highly confident TEIs. These candidates manifest both breakpoints and have reads supporting a target site duplication and a poly-A tail. In addition, TEIs found in two or fewer patients in cohort and showing an MAF under 0.005 or not reported in the gnomAD-SV database were elected as highly presumable insertion candidates. Variants that are in 926,545 of human candidate *cis*-regulatory elements (cCREs) and hearing-loss-associated genes were annotated by retrieving data from the Search Candidate *cis*-Regulatory Elements by ENCODE (SCREEN) database of phase III of the Encyclopedia of DNA Elements (ENCODE) Project^22^. An MAF cutoff of 0.005 and internal frequency were also applied to the selection of *cis*-regulatory element variants as in TEI filtration. In silico prediction of gene perturbation was confirmed using the R package motifbreakR. Throughout the process of selecting candidate variants, the interpretation of clinicians and the inheritance pattern of probands were included. All candidate genetic variants were further confirmed by segregation analysis using Sanger sequencing, PCR or Multiple ligation-dependent probe amplification (MLPA).

## Results

### WGS demonstrated a diagnostic yield comparable to to that of WES

**Fig. 1:**
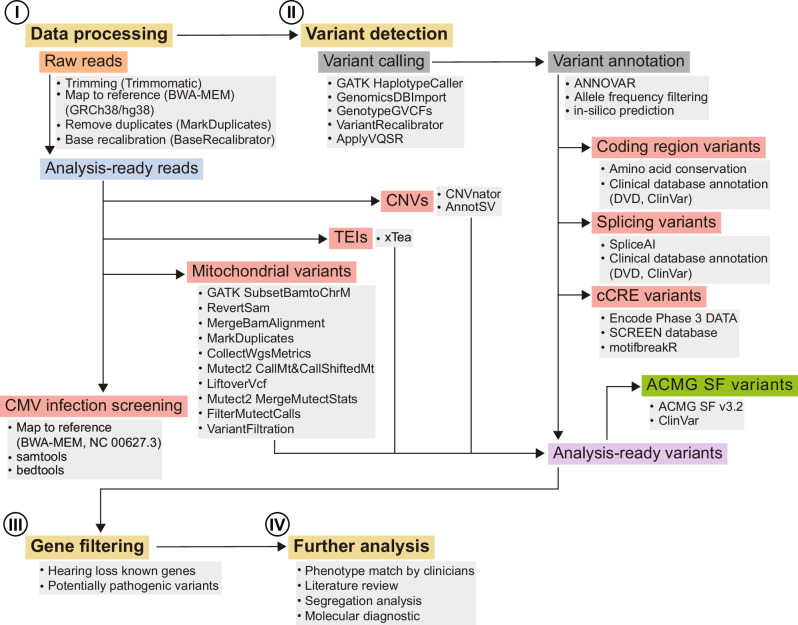
Workflow of WGS analysis. A flowchart illustrating the analysis process of WGS. Coding region variants, splice variants, CNVs, variants in the cCREs, mitochondrial variants, TEI, CMV infection and American College of Medical Genetics and Genomics secondary finding (ACMG SF) variants were detected and analyzed. DVD, Deafness Variation Database.

For the analysis of WGS, we established a multistep analysis process encompassing the detection of coding region variants, cryptic splice variants, *cis*-regulatory element variants, CNVs, mitochondrial variants and TEI (Fig. 1). For the 19 patient samples that underwent both WES and WGS, we conducted a comparison of sequencing depth, gene coverage and variant quality (Supplementary Figs. 1–3). WES demonstrated an average depth of 63× of the kit capture regions, and WGS had an average depth of 37× of the total regions (Supplementary Fig. 1a). For previously reported 173 genes associated with hearing loss (https://hereditaryhearingloss.org/), WES demonstrated an average depth of 53× and WGS had an average depth of 42× (Supplementary Fig. 1b). These targeted hearing-loss-associated loci were covered similarly in both sequencing methods, which was 99.6% (3467/3482) in WES and 99.1% (3449/3482) in WGS (Supplementary Fig. 2). In the WES analysis, about 72% of intervals had 90% of bases or more with depth above 15 (Supplementary Fig. 2a). Meanwhile, in the case of WGS, about 95% of intervals had 90% of bases or more with depth above 15 (Supplementary Fig. 2b). In particular, compared with WGS, a higher proportion of intervals had 60% of bases or less with depth above 15 in WES (Supplementary Fig. 2a, b). Notably, loci demonstrating coverage of 0% were limited to a few exonic regions of *BDP1* in WES and primarily *COL11A2* in WES and WGS. Poor coverage of several regions in *BDP1* and *COL11A2* may be attributed to the presence of adjacent alternative contigs in chromosomes 5 and 6. To assess the quality of SNV called by GATK practices, three main quality parameters for variants, CD, GQ and MRR, retrieved using WGS and WES in hearing-loss-associated loci were compared (Supplementary Fig. 3a–c). Overall, variants called by WGS showed higher and more consistent quality than those called by WES (Supplementary Fig. 3).

**Fig. 2:**
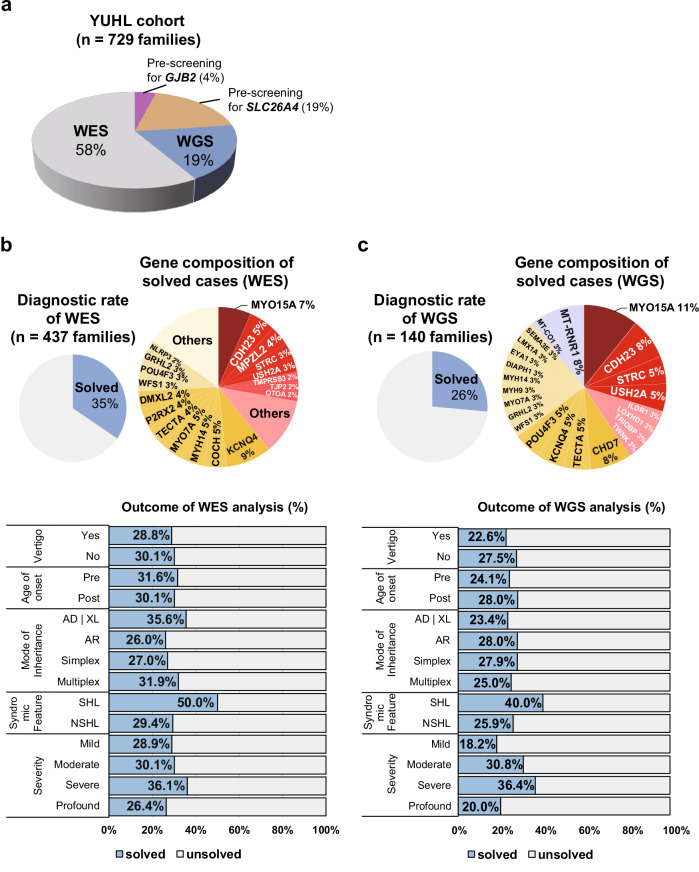
Comprehensive WGS analyses exhibited diagnostic rates comparable to those with WGS for genetic hearing loss. **a** A pie chart representing the distribution of patients subjected to genetic screening in a cohort study. Before next-generation sequencing, patients were prescreened for variants in *GJB2* and *SLC26A4*, the most common genes responsible for hearing loss among the Korean population. **b**, **c** The diagnostic rate and gene composition of patients screened by WES (**b**) and WGS (**c**). Red indicates genes showing an autosomal recessive inheritance pattern, and yellow indicates genes showing an autosomal dominant inheritance pattern. Proportions of diagnosed patients corresponding to clinical manifestations, including vertigo, age of onset, mode of inheritance, syndromic feature and severity of hearing loss, are represented as bar plots; blue, diagnosed; light gray, undiagnosed.

Before conducting genetic testing on patients with hearing loss, we performed a prescreening for pathogenic variants in *GJB2* and *SLC26A4*, the two most prevalent causative genes for nonsyndromic hearing loss in Korean individuals^23,24^. Among the 729 families enrolled in the YUHL cohort, 19% (141/729) of the families harbored pathogenic variants in *SLC26A4* and 4% (30/729) had variants in *GJB2* (Fig. 2a). Out of the 729 families, 437 were referred to WES, while 140 families underwent WGS (Fig. 2a). As mentioned earlier, 19 individuals underwent both WES and WGS. In the case of WES, diagnostic yield was approximately 35% (151/437) of cases (Fig. 2b). The frequent causative genes for families exhibiting an autosomal recessive inheritance pattern were *MYO15A* (10/437), *CDH23* (8/437) and *MPZL2* (6/437). Meanwhile, *KCNQ4* (14/437), *COCH* (7/437), *MYH14* (7/437) and *MYO7A* (7/437) were common causes of families showing an autosomal dominant inheritance pattern (Fig. 2b). The diagnostic yield was similar regardless of the presence of vertigo or the age of onset. However, patients with a family history of multiple affected individuals, autosomal dominant inheritance or severe or syndromic hearing loss demonstrated a higher rate of successful diagnoses (Fig. 2b).

The diagnostic rate of WGS was approximately 26% (37/140) (Fig. 2c). The diagnostic rate seen in our cohort was lower for WGS compared with WES. However, it was challenging to directly compare the diagnostic rates between WES and WGS owing to considerable differences in the patient characteristics recruited for each sequencing method (Supplementary Table 1). The most frequent causative gene for families exhibiting an autosomal recessive inheritance pattern was *MYO15A* (4/140). Meanwhile, *CHD7* (3/140), *TECTA* (2/140), *KCNQ4* (2/140) and *POU4F3* (2/140) were detected in families showing an autosomal dominant inheritance pattern (Fig. 2c and Supplementary Table 2). Interestingly, some patients who underwent WGS were identified to have pathogenic CNVs (2/140), a cryptic splice variant (2/140) and mtDNA variants (4/140), which are difficult to detect with WES. Patients who exhibited postlingual, autosomal recessive inheritance, severe hearing loss or syndromic hearing loss rather than nonsyndromic hearing loss showed higher diagnostic rates with WGS (Fig. 2c).

### Aberrant splice variants located outside the core splice sites were identified through WGS

For patients referred to WGS, a comprehensive analysis of splicing variant was conducted because WGS covers a broader intergenic and intronic region of the genome, rather than being restricted to exon-flanking intronic regions^25^. SpliceAI, a 32-layer deep neural network that predicts splicing from a pre-mRNA sequence, was utilized for the detection of potential cryptic splice variants^26^. We filtered and prioritized the detected splice variants that had a delta score higher than 0.1, indicating a high probability of splice alteration (Supplementary Table 3).

**Fig. 3:**
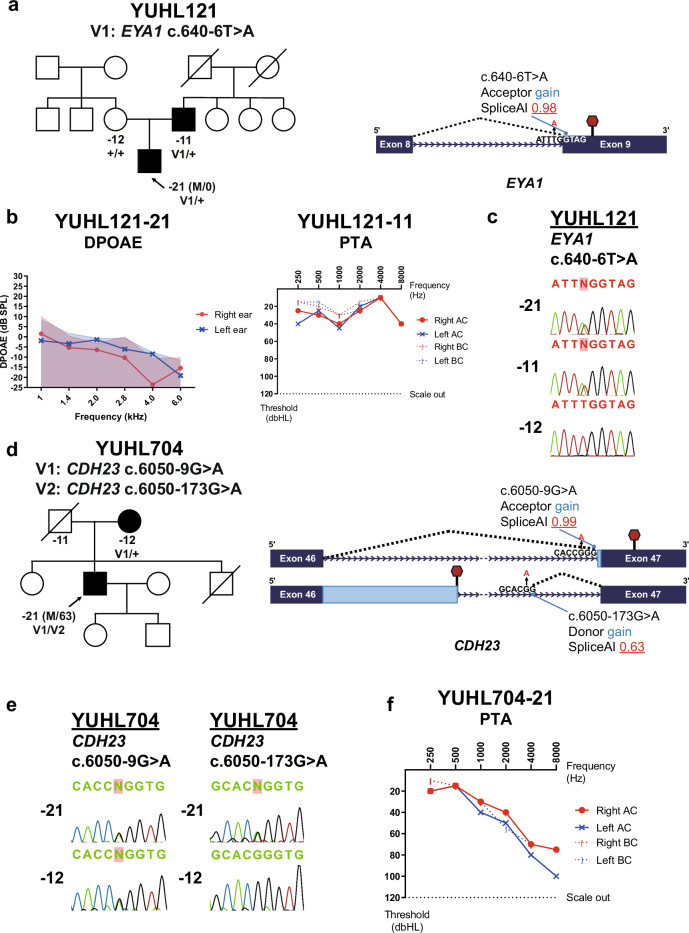
Deep-learning-based splicing prediction of WGS identified cryptic splice mutations outside of core splice site. **a** A pedigree of proband YUHL121-21 and graphical scheme of predicted gene perturbation by aberrant splicing variant, c.640-6T>A of *EYA1*. The altered allele is labeled in red, and the predicted probability of variant acting as a splice acceptor is indicated; red hexagon, premature stop codon. **b** Distortion-product otoacoustic emission (DPOAE) (left) and pure-tone audiometry (PTA) (right) results of YUHL121-21 and YUHL121-11, respectively. For DPOAE, dashed lines indicate the noise floor, and solid lines indicate detected distortion products. AC, air conduction thresholds; BC, bone conduction thresholds. **c** Segregation results of a c.640-6T>A variant in YUHL121 family. Sanger sequencing chromatograms revealed that c.640-6T>A from was inherited from the father of a proband. -21, proband; -11, father; -12, mother. **d** A pedigree of the YUHL704 family and a graphical scheme of the predicted gene perturbations by aberrant splicing variants in the *CDH23* gene. The altered allele is labeled in red, and the predicted probability of variants acting as cryptic splice acceptor or donor is indicated; red hexagon, premature stop codon. **e** Segregation results of the c.6050-9G>A and the c.6050-173G>A variants in the YUHL704 family. -21, proband; -12, mother. **f** PTA results of YUHL704-21. AC, air conduction thresholds; BC, bone conduction thresholds.

In the YUHL121 family, we identified the c.640-6T>A variant in the eighth intron of *EYA1* in patient YUHL121-21 through WGS (Fig. 3a). The region harboring the variant was covered by both WES and WGS, although the patient was screened only by WGS. Variants in *EYA1* are the most common cause of branchio-oto-renal syndrome, typically inherited in an autosomal dominant manner^27^. The proband (YUHL121-21) was a 10-month-old male referred for bilateral congenital moderate hearing loss. The click Auditory brainstem response (ABR) test showed thresholds of 60 dB for both ears, and the distortion-product otoacoustic emission (DPOAE) test was negative for both ears (Fig. 3b). He had a family history of hearing loss in his father, who exhibited mild conductive hearing loss in mid-to-low frequencies (Fig. 3b). Clinical examination revealed bilateral preauricular pits without other external ear anomalies. The proband had previously undergone excisional surgery for bilateral second branchial cleft cyst at another hospital 3 months before visiting our clinics. Genitourinary ultrasonography showed no evidence of renal anomalies. Based on these clinical features, the clinician suspected branchio-oto-renal syndrome according to the diagnostic criteria proposed by Chang et al.^28^. Segregation analysis by Sanger sequencing in the YUHL121 family verified the variant and revealed that the heterozygous variant was inherited from the father (Fig. 3c). Furthermore, the detected variant was never reported in the population, according to the Genome Aggregation Database (gnomAD), and predicted to cause aberrant mRNA splicing, creating a premature terminating codon and truncation of the C-terminal EYA domain, where most of the reported variants are clustered (Fig. 3a)^29^. In addition, the probability of being loss-of-function intolerant (pLI) score of the *EYA1* gene is 1.0 according to the gnomAD, indicating that this gene is highly likely to be haploinsufficient. In summary, co-segregation within an affected family member and the molecular characteristics, combined with evident clinical phenotypes, led us to classify the c.640-6T>A variant as potentially pathogenic.

In the YUHL704 family, we identified compound heterozygous variants of the *CDH23* gene, the c.6050-9G>A near the 47th exon and a deep-intronic c.6050-173G>A variant between the 46th and 47th exons through WGS (Fig. 3d). The c.6050-9G>A variant was in a region covered by both WES and WGS, whereas the c.6050-173G>A variant showed poor coverage or no coverage at all by WES. The c.6050-9G>A variant was previously reported as pathogenic in several studies^30,31^. This variant is predicted to create a cryptic splicing acceptor, inserting seven additional nucleotides from the 46th intron and introducing a premature stop codon in the following exon, resulting in nonsense-mediated decay of the transcript^30^. Furthermore, the c.6050-173G>A variant in the deep-intronic region of 46th intron is predicted to create a cryptic splicing donor, which might induce the extension of the 46th exon and the introduction of a premature termination codon. Moreover, this variant has never been reported in gnomAD. Segregation analysis by Sanger sequencing of the YUHL704 family revealed that the c.6050-9G>A variant was inherited from the mother, while the c.6050-173G>A variant was not detected in her, indicating that the two variants are in a *trans* state (Fig. 3e). The proband was a 63-year-old male and exhibited progressive hearing loss initiated in his 20s without definite vestibular dysfunction. Audiometric evaluation indicated bilateral, moderate, ski-sloping hearing loss (Fig. 3f). These clinical characteristics are consistent with DFNB12, caused by variants in the *CDH23* gene and presenting with a milder phenotype compared with type 1D Usher syndrome^32^. Therefore, we concluded that the identified variants potentially explain his hearing loss, which would otherwise be unexplainable by WES owing to the hidden deep-intronic variant.

### WGS detected pathogenic mtDNA mutations

**Fig. 4:**
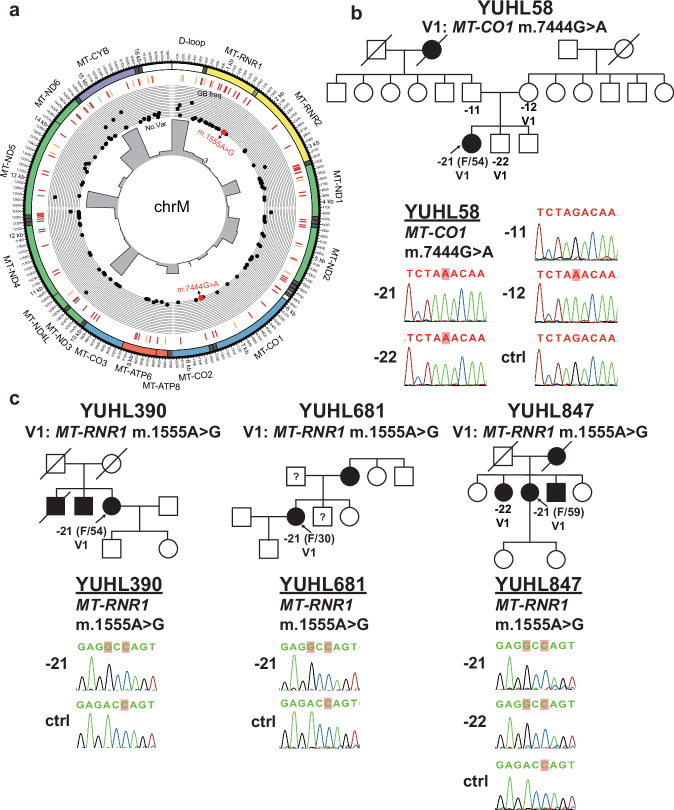
Pathogenic homoplasmic m.1555A>G and m.7444G>A were responsible for maternally inherited hearing loss. **a** A Circos plot of the mitochondrial genome. The scatter plot and heat map represent the retrieved GenBank frequency of 119 variants among total detected mitochondrial variants in patients screened by WGS. Putative pathogenic variants, m.1555A>G and m.7444G>A, are labeled in red. The histogram represents the number of patients harboring variants present in a region when the chromosome is divided into 500-bp bins. **b** A pedigree of proband YUHL58-21 and segregation results of a m.7444G>A variant in the YUHL58 family. Sanger sequencing chromatograms revealed that homoplasmic m.7444G>A was maternally inherited. -21, proband; -11, father; -12, mother; -22, sibling; ctrl, control. **c** A pedigree of proband YUHL390-21, YUHL681-21 and YUHL847-21 and segregation results of a m.1555A>G variant in the corresponding family. Detected homoplasmic m.1555A>G are further confirmed by Sanger sequencing. -21, proband; -22, sibling.

To eliminate sequencer noise and contamination of inserted mtDNA into the nuclear genome or noncoding control region, we followed the best practices of the mitochondrial short-variant discovery pipeline of GATK (Fig. 1) for the discovery of variants in mitochondria DNA. Due to the limited capacity of WES in detecting mitochondrial variants^19^, only WGS samples were used for variant discovery. Initially, a total of 1338 mtDNA variants were identified through the analysis. Among them, allele frequencies for 119 mtDNA variants were available from GenBank and a few variants previously reported to be associated with hearing loss were detected in our cohort (Fig. 4a and Supplementary Table 4). Upon thorough review of the clinical history and inspection of copies of detected mtDNA variants, hearing loss in a patient (YUHL58-21) carrying the homoplasmic m.7444G>A mutation in *MT-CO1*, and in three patients (YUHL390-21, YUHL681-21 and YUHL847-21) harboring the homoplasmic m.1555A>G mutation in *MT-RNR1*, was determined to be associated with mtDNA variants (Fig. 4b, c).

The m.7444G>A variant has been causally linked to deafness in several studies and has also been assumed to influence the penetrance and expressivity of the m.1555A>G mutation^33,34^. In addition, the m.1555A>G mutation is commonly reported to be associated with nonsyndromic and aminoglycoside-induced hearing loss^35^. The clinical characteristics of the four patients identified with the m.1555A>G and m.7444G>A mutations are summarized in Supplementary Table 5. Both the m.7444G>A and m.1555A>G mutations lead to hearing loss with variable expressivity^36,37^. Consistently, the four patients exhibited heterogeneous phenotypes, ranging from bilateral, congenital and profound hearing loss to asymmetric, postlingual or mild hearing loss. None of the patients reported exposure to aminoglycosides, although an accurate record of administration was impossible due to the retrospective nature of the history interview. The hearing loss in YUHL390, YUHL681 and YUHL847 was familial, and that in YUHL681 and YUHL847 was suspected to be maternally transmitted, although incomplete penetrance was observed. Interestingly, the mother of YUHL681-21 reported that her hearing loss had commenced after the administration of antibiotics for a bacterial infection, suggesting the possibility of aminoglycoside-induced hearing loss. No other syndromic symptoms were observed in any of the four patients. Segregation analysis by Sanger sequencing confirmed that the m.7444G>A variant from YUHL58 was inherited from her mother (YUHL58-12), and her brother (YUHL58-22) carried an identical mtDNA variant, suggesting maternal inheritance (Fig. 4b). Furthermore, the presence of the m.1555A>G variant in the three patients and its co-segregation in a sister (YUHL847-22) of YUHL847-21 were confirmed by segregation analysis (Fig. 4c). To further determine the genetic cause of hearing loss in these four families, we also examined other variants in hearing-loss-associated genes detected by WGS and found no pathogenic or likely pathogenic variants other than m.7444G>A or m.1555A>G variant that could plausibly explain their hearing loss. Therefore, WGS revealed deafness-causing mtDNA variants, which are typically undetectable by WES.

### Read-depth-based CNV analysis of WGS revealed the presence of a biallelic gross deletion and a pathogenic variant

**Fig. 5:**
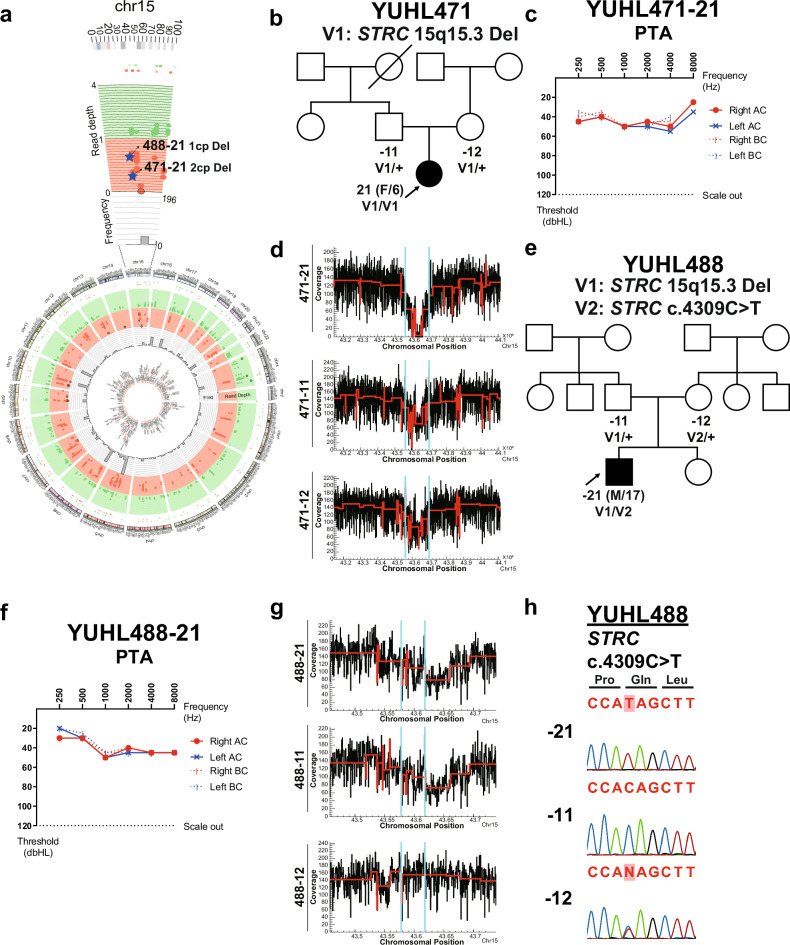
WGS revealed that a gross deletion in *STRC* led to early-onset, moderate and recessive hearing loss. **a** A Circos plot of the human whole genome and a magnified region of chromosome 15 including the *STRC* gene. The innermost part of the circle represents genes associated with hearing loss. The scatter plot and heat map represent the read depth of detected CNVs from the analysis. Green color represents duplication, and red color represents deletion. Putative pathogenic homozygous deletion of YUHL471-21 and heterozygous deletion of YUHL488-21 in the *STRC* region are plotted as blue stars. The histogram represents the number of patients harboring variants present in a region when the chromosome is divided into 2,000-kbp bins. **b**, **c** A pedigree (**b**) and PTA result (**c**) of YUHL471-21. AC, air conduction thresholds; BC, bone conduction thresholds. **d** Visualization of read-depth (RD) signals of detected homozygous deletion in the YUHL471 family. The deleted region is bordered by two light-blue lines. The red line indicates average read-depth signals spanning a given chromosomal position. **e**, **f** A pedigree (**e**) and PTA result (**f**) of YUHL488-21. AC, air conduction thresholds; BC, bone conduction thresholds. **g** Visualization of RD signals of detected heterozygous deletion in the YUHL488 family. The deleted region is bordered by two light-blue lines. The red line indicates average read-depth signals spanning a given chromosomal position. Heterozygous deletion in the *STRC* gene was paternally inherited. **h** Segregation results of a c.4309C>T variant in the YUHL488 family. Sanger sequencing chromatograms revealed that heterozygous c.4309C>T was inherited from the mother of a proband and c.4309C>T was sequenced as a hemizygote in the proband due to a heterozygous deletion inherited from the father. -21, proband; -11, father; -12, mother.

CNVs are suspected as an underrecognized cause of nonsyndromic hearing loss^38^. Because the sensitivity and coverage, essential for CNV discovery, of WGS have been previously reported to surpass those of WES^39^, we comprehensively analyzed CNVs within hearing-loss-associated loci using WGS (Supplementary Fig. 4). We identified several gross deletions in causative genes for nonsyndromic hearing loss, including *STRC*, *COL1A1*, *CHD7*, *PTPRQ*, *SLC12A2* and *ABHD12* (Supplementary Table 6). Among these, pathogenic CNVs in chromosomal position 15q15.3, known as the most common loci harboring long deletions or CNVs in patients with nonsyndromic hearing loss^40^, were identified in YUHL471-21 and YUHL488-21 (Fig. 5a).

YUHL471-21 was a 6-year-old female with hearing loss that began between the ages of 2 and 3 years. The family history indicated that her hearing loss was autosomal recessive, and pure-tone audiometry revealed bilateral, moderate hearing loss with a flat configuration (Fig. 5b, c). We conducted WGS for this patient to identify the molecular cause of her hearing loss, and read-depth-based CNV analysis by CNVnator revealed homozygous deletions of the *STRC* and *CATSPER2* genes in YUHL471-21 (Fig. 5d and Supplementary Fig. 5). Nucleotide resolution of breakpoints was apparent in this complete deletion region (Supplementary Fig. 5). Moreover, heterozygous deletion of the identical region in her parents (YUHL471-11 and YUHL471-12) was also confirmed on the basis of the read-depth signal (Fig. 5d and Supplementary Fig. 5). Based on the results of trio-analysis and typical clinical characteristics of *STRC*-associated hearing loss, which is the most common cause of moderate, flat-configured hearing loss in Korean individuals^41^, we determined that the hearing loss of YUHL471-21 was attributable to the homozygous deletion of *STRC*.

Another patient, YUHL488-21, detected with a CNV of *STRC*, was a 17-year-old male referred for hearing loss that started in his early teenage years. His hearing loss appeared to be autosomal recessive, and audiologic examination showed bilateral, moderate and flat-configured SNHL (Fig. 5e, f). YUHL488-21 had an extensive 26,000-bp heterozygous deletion in the *CKMT1B*-*STRC* gene, and the deletion in the same region was also detected in the father of the proband, YUHL488-11 (Fig. 5g and Supplementary Table 6). The read-depth estimated by the CNV detection tool confirmed that this deletion was heterozygous in YUHL488-21 and 488-11, compared with the mother who did not have this CNV (Fig. 5g and Supplementary Fig. 5). Interestingly, the nonsense variant, c.4309C>T, located in the aforementioned region of deletion in *STRC*, was detected as hemizygous in the proband (Fig. 5e, h). Subsequent segregation analysis revealed that this variant was detected as heterozygous in the mother, YUHL488-12 (Fig. 5h), and was maternally inherited. The c.4309C>T variant was reported to be rare (with an allele frequency of 0.000004959, according to gnomAD), and it creates a stop codon at a conserved glutamine residue, resulting in a truncated protein that is 339 amino acids shorter than the full-length protein and may impair its normal function. Considering the autosomal recessive inheritance pattern of hearing loss within the family, the stereotypic clinical characteristics of *STRC*-related hearing loss^42^, and the molecular characteristics of both variants, the proband’s hearing loss probably resulted from a concurrent nonsense mutation (c.4309C>T) and heterozygous copy number deletion in the *STRC* gene.

### The random insertion of TEs into a gene may contribute to the disruption of its function

TEs, particularly retrotransposons, are segments of DNA that can propagate within the genome and are largely divided into three groups: long interspersed nuclear elements 1 (L1), Alu and SINE-VNTR-Alu^43^. TEs have biological significance by potentially acting as insertional mutagens and perturbing the arrangement of DNA, resulting in the alteration of gene expression or RNA splicing^44,45^. In this regard, we thoroughly inspected the insertion of TEs in known hearing-loss-associated loci using WGS samples. After removing concurrent intersample TEs and considering allele frequencies retrieved from the population database, we finally selected several potentially detrimental insertions of TEs in *SLC17A8*, *CDH23*, *EYA1*, *PAX3* and *TMC1* (Supplementary Table 7).

**Fig. 6:**
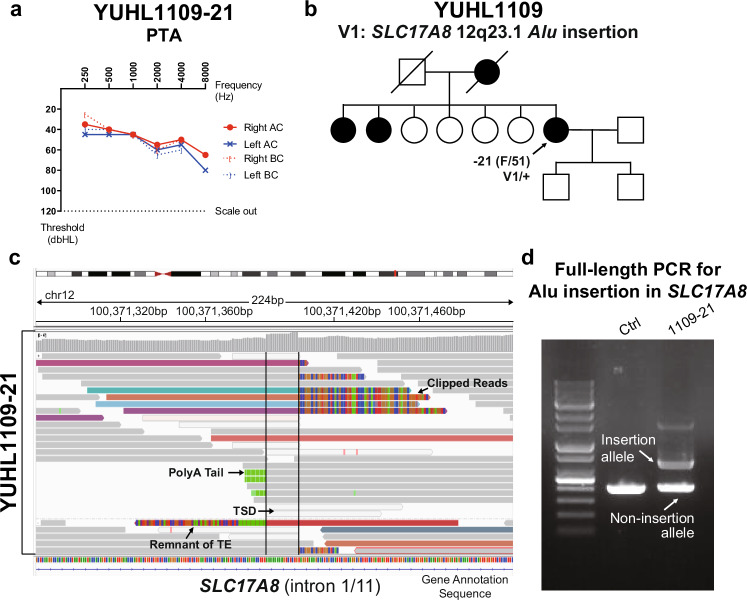
TE Alu insertion was identified in *SLC17A8* of patients with hearing loss by WGS screening. **a**, **b** PTA results (**a**) and a pedigree (**b**) of YUHL1109-21, AC, air conduction thresholds; BC, bone conduction thresholds. **c** An Integrative Genomics Viewer image at the insertion site in gene *SLC17A8* in YUHL1109-21. The sequencing coverage (top) and sequencing reads (bottom) are shown. The insertion shows the canonical signatures of target-primed reverse transcription (TPRT)-mediated retrotransposition: 15-bp target site duplication (TSD) between the two insertion breakpoints, a poly-A tail, supporting clipped reads and discordant reads with mates mapping to the consensus Alu sequence. **d** Full-length PCR validation of the Alu insertion in *SLC17A8* in YUHL1109-21. In blood-derived genomic DNA, we validated only the insertions in the proband, compared with the nonaffected control.

Among the identified patients, YUHL1109-21 was a 51-year-old female with progressive and moderate SNHL in both ears that started in her early 30s (Fig. 6a). Her mother and brothers also had hearing loss, suggesting an autosomal dominant inheritance pattern (Fig. 6b). We conducted WGS to identify the molecular cause of her hearing loss, and no pathogenic or likely pathogenic variants were identified in the coding regions of hearing-loss-associated genes. However, in-depth analysis of WGS data to identify TEIs discovered chimeric reads at the chr12:100371388 (GRCh38) position due to a heterozygous Alu insertion in the 1st intron of the *SLC17A8* gene in the proband, characterized by nearby soft-clipped reads, unmapped poly-A tails and targeted-site duplications (Fig. 6c). According to the gnomAD-SV database, this insertion has not been documented in the general population. When the genomic region of the TEI was amplified by PCR, an abnormal PCR product with a larger size than the normal product was observed in YUHL1109-21 (Fig. 6d), corroborating the possibility of a heterozygous Alu insertion. Although the postlingual, progressive and high-frequency affected hearing loss of YUHL1109-21 was consistent with previous reports of *SLC17A8*-associated hearing loss^46^, whether this Alu insertion in the *SLC17A8* gene functionally contributes to the disease requires further investigation.

### Mutations in *cis*-regulatory elements might contribute to nonsyndromic hearing loss

The precise spatiotemporal regulation of gene expression plays a critical role in the normal development of the inner ear from the otocyst^47^. Therefore, we hypothesized that disruptions in the timely orchestrated regulation of gene expression, stemming from overlooked variants in *cis*-regulatory elements of genes associated with hearing loss, could potentially contribute to the condition. Utilizing data from phase III of the ENCODE Project^22^, we screened for variants that could potentially affect the expression of known hearing-loss-associated genes, among 926,535 human cCREs from the SCREEN database^22^. Among these elements, we identified six potential variants in *cis*-regulatory elements associated with hearing loss genes, *CDH23*, *OTOF*, *MYO6*, *COL4A4* and *WHRN* (Supplementary Table 8). Considering the inheritance pattern and clinical phenotypes, we determined that these variants are insufficient to be classified as pathogenic. Nevertheless, our results emphasize the need to identify potential mutations in regulatory regions of known hearing loss genes and validate their effects on the elaborate regulation of inner-ear-specific transcription factors to reveal unknown causes of hearing loss.

### Secondary findings in patients with deafness by WGS

In addition to genetic factors, congenital CMV infection is also one of the leading causes of SNHL and neurodevelopmental complications^48^. Generally, newborns are screened for CMV infection by PCR of CMV DNA in urine or saliva, but a recent study detected CMV infection by utilizing WGS from blood specimens^49^. Therefore, we screened for potential CMV infection in our patients with hearing loss who underwent WGS. Consequently, we found that a significant number of reads were aligned to the CMV genome among 1.4% (2/140) of patients, suggesting a history of CMV infection (Supplementary Table 9). However, both identified patients already carried causative pathogenic variants in *LOXHD1* and *KCNQ4* attributable to their hearing loss (Supplementary Table 9). Because hearing loss arises from congenital CMV infection, in contrast to postnatal CMV infection, which is not recognized as a direct cause of SNHL in healthy individuals^50^, and WGS cannot determine the exact period of infection, we could not ascertain that the identified evidence of CMV infection from WGS is the cause of their hearing loss. However, these cases suggest that WGS can provide additional valuable information on one of the overlooked causes of SNHL, alongside genetic etiologies.

Recently, the American College of Medical Genetics and Genomics published guidance for reporting secondary findings in the context of clinical exome and genome sequencing^51^. For opportunistic screening to identify and manage additional risks for specific genetic disorders not just limited to SNHL and offer established interventions to patients, we detected variants of these listed genes classified as pathogenic or likely pathogenic in the clinical database. This approach detected several pathogenic variants in *MUTYH*, *MSH2*, *TTN*, *DES*, *BTD*, *BRCA2*, *ATP7B*, *TP53*, *GAA* and *LDLR* genes (Supplementary Table 10). Most of variants were loss-of-function variants perturbing protein, such as nonsense, frameshift and aberrant splice variants, located in obligatory splicing sites. Interestingly, the c.744+2del variant in *MSH2* was highly frequent in our cohort. Further clinical interpretation of these secondary findings is required for critical discussions with clinicians considering ethnicity-specific frequencies of allele, inheritance mode and phenotype of patients.

## Discussion

In recent years, next-generation sequencing has undergone substantial advances, proving its diagnostic value in unveiling the genetic etiologies of multiple disease^52^. In the realm of hereditary hearing loss, multiple studies utilizing high-throughput WES have identified key causative genes contributing significantly to hearing loss^11–16^. However, despite the advantages of WES, over 60% of patients with Mendelian inheritance patterns of hearing loss remain undiagnosed, lacking precise molecular characterization (Fig. 1). This lack of conclusive genetic diagnoses arises from various factors, including ambiguous genotype–phenotype correlations, insufficient evidence supporting identified variants, incomplete penetrance, variable expressivity or allelic heterogeneity in hearing loss^17^. Moreover, from a technical perspective, WES may fail to detect variants located outside coding regions, such as those in intergenic or deep-intronic regions, *cis*-regulatory regions or mtDNA, and it may not be suitable for sensitively identifying CNVs. Indeed, recent study in families with rare monogenic diseases suggested that approximately 8% (61/744) of diagnoses were missed by exome sequencing, but when the genetic screening method was expanded to WGS, the diagnostic yield of those negative evaluations increased by 2.3% (17/744)^53^. This result highlights the need to utilize platforms such as short-read genome sequencing or even long-read genome sequencing for the diagnosis of variants that may elude exome sequencing. Accordingly, to address limitations of WES or targeted sequencing and elucidate the genetic etiologies of unresolved cases by WES, we conducted extensive WGS analyses on patients with nonsyndromic hearing loss and established a systematic pipeline for investigating pathogenic genetic alterations throughout the entire genome.

The discovery of putative pathogenic intronic variants that disrupt normal splicing was one of the important findings from our study. During variant discovery using WES, variants located outside of obligatory splicing sites are frequently overlooked. However, those variants located in deep-intronic regions missed by WES sometimes significantly contribute to Mendelian disorders^54^. Furthermore, splicing-affecting variants located in deep-intronic regions are more amenable targets of splice-switching oligonucleotide therapy than those in canonical splicing sites^55^. Therefore, it is imperative to identify hidden splicing-disruptive variants commonly underdetected by WES with WGS in terms of actionability for the development of novel therapeutics for hearing loss.

Although less prevalent, there is a growing body of studies reporting hearing loss caused by mtDNA variants, which account for approximately 1.5–5% of hearing loss cases, depending on ethnicity^37^. Notably, the clinical characteristics of hearing loss caused by mtDNA variants are highly heterogeneous, and the family history of affected patients is often inconclusive due to incomplete penetrance^37^. Therefore, a systemic approach using WGS holds implications for discovering unsuspected and undetected mtDNA variants, particularly when the diagnosis relies solely on clinical findings and WES. Furthermore, because the m.1555A>G variant, the most prevalent mtDNA variant associated with hearing loss, is linked to augmented aminoglycoside-related ototoxicity^35^, accurate identification of hearing-loss-related mtDNA variants carries clinical significance and can guide physicians in preventing further disease exacerbation.

CNVs have been reported as a significant cause of multiple Mendelian diseases^56^, including nonsyndromic hearing loss^38^. Especially for hearing loss, *STRC* is the most frequently identified causative gene with a segmental deletion of DFNB16 locus, sometimes accompanied by a deletion of adjacent *CATSPER2* gene resulting in deafness–infertility syndrome^57^. Here, we identified homozygous deletion and heterozygous deletion with a hemizygous nonsense mutation in the *STRC* gene and successfully mapped the exact breakpoint of deletion with WGS data. The mapping of breakpoints of CNVs can enable the interpretation of structural complexity and pathogenicity of identified CNVs, which is challenging with WES data providing only low-resolution information due to limited genome coverage and depth^58^. In particular, the aforementioned hemizygous nonsense variant was initially not detected in WES but was identified upon reexamining the data with WGS. Furthermore, besides deletions in the *STRC* gene, CNVs in other deafness-causative genes, such as *OTOA* and *TMC1*, have been reported^38^, underscoring the importance of identifying CNVs and interpreting their impact on the pathogenesis of the disease.

The inner ear is a complex organ comprising highly heterogeneous cell types, each differing in morphology, functional role and anatomical localization. The tightly orchestrated differentiation and development of these various cell types are governed by multiple key transcription factors dictating gene regulatory networks in a timely manner^47,59^. However, while the identification of variants in *cis*-regulatory regions has been reported in multiple other diseases^60^, their exact contribution to hearing loss remains largely unknown. Therefore, the detection of variants in *cis*-regulatory regions of multiple deafness-causative genes in our study underscores the need for multi-omics analysis to elucidate the pathogenic role of these variants in hearing loss. Although our study did not identify any pathogenic cCRE variants, we assert that a more comprehensive analysis of the association between these cCRE variants and hearing loss phenotypes, utilizing approaches such as burden test or association-based analysis, is necessary, contingent upon the availability of proper control.

Recently, TEs, particularly retrotransposons, have been increasingly recognized as significant contributors to Mendelian diseases. Furthermore, through WGS, detrimental insertions of TE into deep-intronic regions have been reported, and their impacts on normal splicing and therapeutic implications have also been unveiled^44,61^. In this study, we identified a hidden Alu insertion in the *SLC17A8* gene by WGS in a patient with hearing loss otherwise unexplained by WES. A more accurate estimation of the contribution of mobile element insertion to hearing loss requires further analysis with a larger cohort.

In addition to the identification of potential candidate variants in hearing-loss-associated genes, we also found a few deleterious variants in a list of genes with secondary findings in clinical exome and genome sequencing provided by the American College of Medical Genetics and Genomics^51^. These secondary findings might provide additional information to the patients of medically actionable variants and can be useful within the context of optimizing potential medical benefit to the patient, even when they are unrelated to the primary medical reason for testing.

Nevertheless, this study has several limitations. Although WGS partially addressed undiagnosed cases missed by WES, the overall diagnostic rate of WGS did not exhibit a significant increase compared with that of WES. This outcome may stem from the differences in the patient populations undergoing WES versus WGS. Variations in the onset, severity and mode of inheritance of hearing loss were observed between patients subjected to WES and those undergoing WGS. Patients undergoing WES displayed a higher frequency of postlingual, autosomal dominant and multiplex hearing loss cases, potentially impacting the diagnostic yield. In addition, this study did not functionally confirm the contribution of TEIs to the pathology. Further investigations, such as minigene assays to evaluate the impact of mobile element insertions on normal splicing, and transcriptomic analyses^62^ to assess the consequences of variants on gene regulatory networks of specific cell types within the inner ear, are necessary to determine whether these variants indeed induce hearing loss.

Overall, this study presented previously overlooked genetic etiologies of hearing loss through a comprehensive systemic analysis based on WGS. In addition to coding region variants, intronic variants disrupting normal splicing, mtDNA variants and CNVs were identified in patients with nonsyndromic hearing loss unresolved by WES. Furthermore, TEIs potentially contributing to hearing loss were uncovered. Through a thorough investigation into the molecular basis of hearing loss with WGS, a complete genetic landscape of the condition will be unveiled. Furthermore, based on these findings, the development of patient-customized therapeutics will become feasible.

## Supplementary information

Supplementary Information

## Data Availability

The data that support the findings of this study are available from the corresponding author upon reasonable request.
