# Supplementary material for: Systematic genetic assessment of hearing loss using whole-genome sequencing identifies pathogenic variants
Source: Exp Mol Med. 2025 Apr 1;57(4):775–87. doi: 10.1038/s12276-025-01428-x (PMC12046045; doi:10.1038/s12276-025-01428-x)
Supplement: Supplementary file 1 — Supplementary Information [file 12276_2025_1428_MOESM1_ESM.pdf]

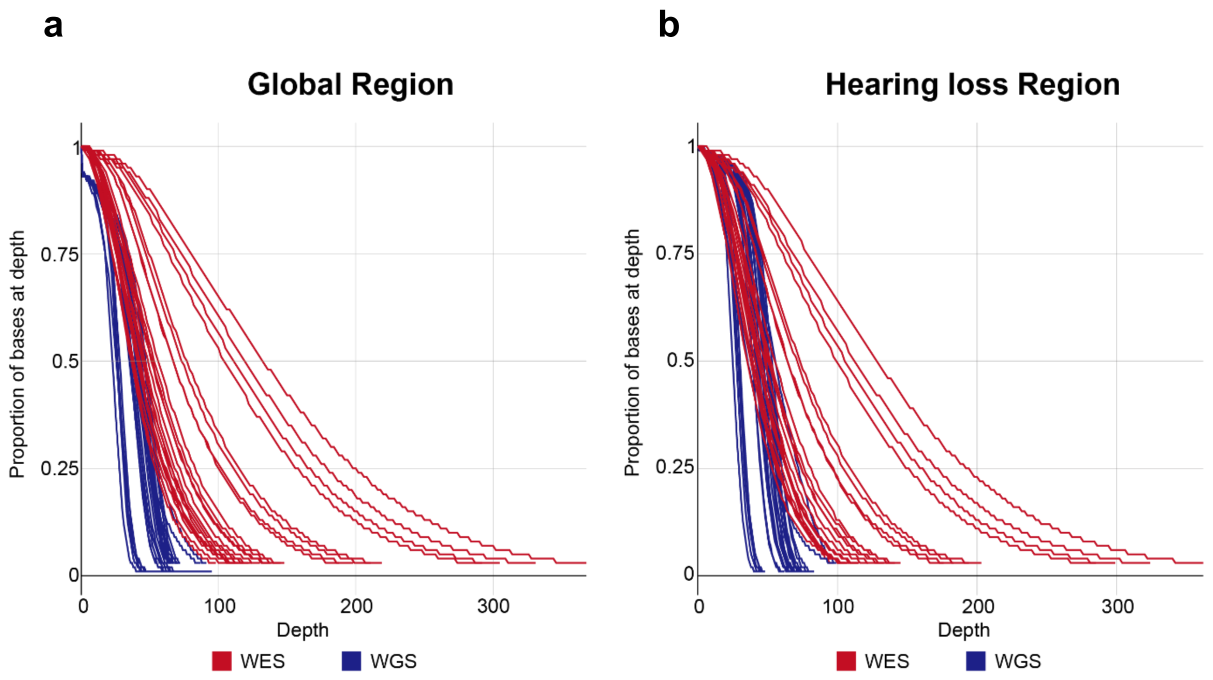

Supplementary Fig. 1. Sequencing depth of samples both screened by WES and WGS

a Sequencing depth of samples screened by WES and WGS over entire genomic region and **b** genomic regions associated with hearing loss. As of WES, an Agilent SureSelect V5 enrichment capture bed was given as interval options and the depth of corresponding regions was calculated, blue; WGS, red; WES

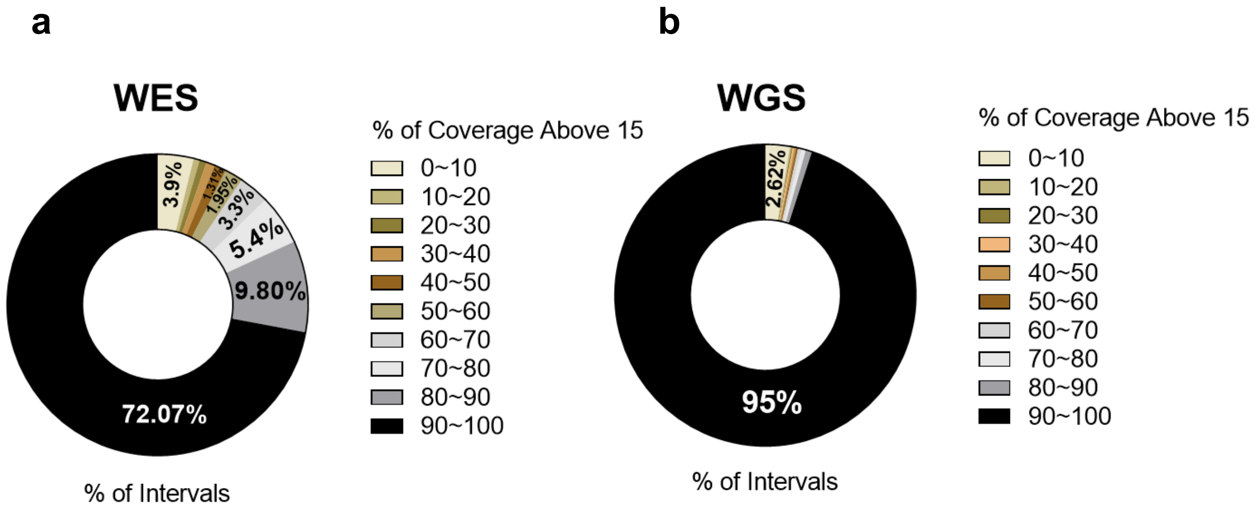

Supplementary Fig. 2. Coverage of genes associated with hearing loss in WES and WGS

A pie plot representing the distribution of percentage of intervals corresponding to certain coverage by WES (**a**) and WGS (**b**) over regions associated with hearing loss. Detailed methods of coverage calculations are described in the method section.

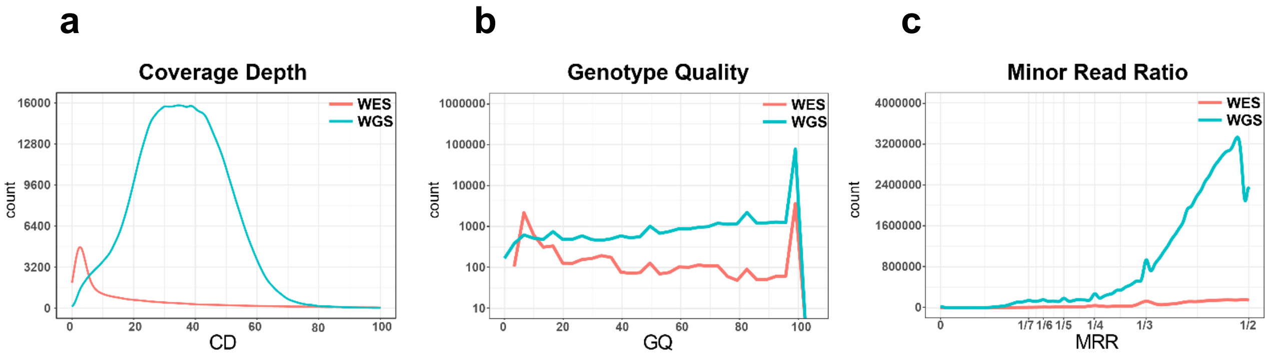

Supplementary Fig. 3. Distribution of the three main quality parameters for the variants detected by WES or WGS in genes associated with hearing loss

Distribution of the three main quality parameters for the variants in hearing loss associated regions detected by WES or WGS. (**a**) Coverage depth (CD), (**b**) genotype quality (GQ) score, and (**c**) minor-read ratio (MRR). Coverage depth (CD), corresponding to the number of aligned reads covering a single position; and genotype quality (GQ), indicates accuracy of genotype calls. The minor-read ratio (MRR) is defined as the ratio of reads for the less covered allele (reference or variant allele) over the total number of reads covering the position at which the variant was called. For each of the three parameters, the average of samples both undergone WES (red) and the six WGS (turquoise) were plotted.

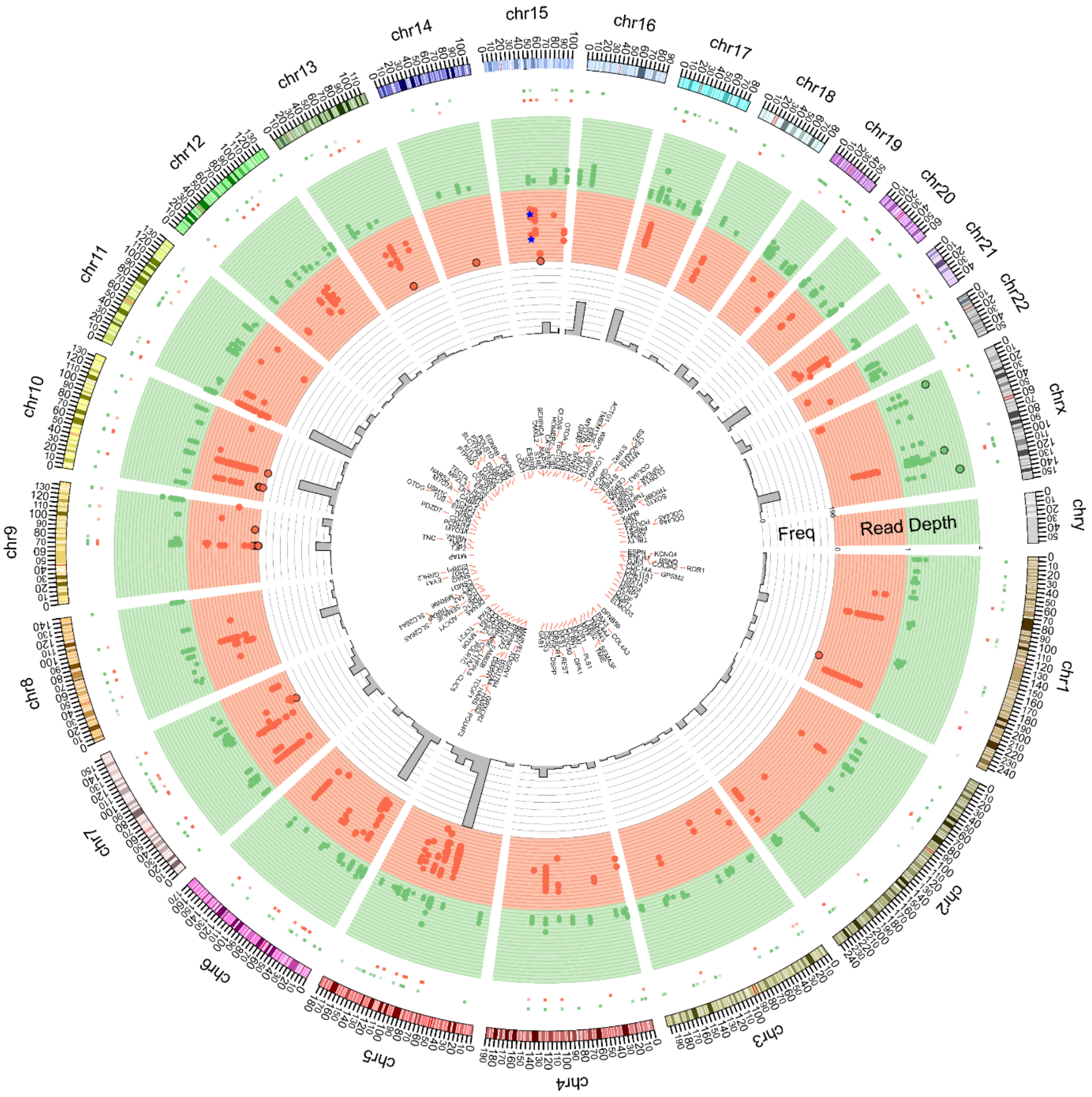

Supplementary Fig. 4. Genome-wide circos plot of total detected CNVs of WGS samples

A circos plot of human whole genome with total detected copy number variants (CNVs) in the study. The innermost part of the circle represents genes associated with hearing loss. Scatter plot and heatmap represent read depth of total detected CNVs from the analysis. Green color represents duplication and red color represents deletion, respectively. Putative pathogenic homozygous deletion of YUHL471-21 and heterozygous deletion of YUHL488-21 in *STRC* region are plotted as blue star. A histogram represents the number of patients harboring variants present in a region when the chromosome is divided into 2,000kbp bins.

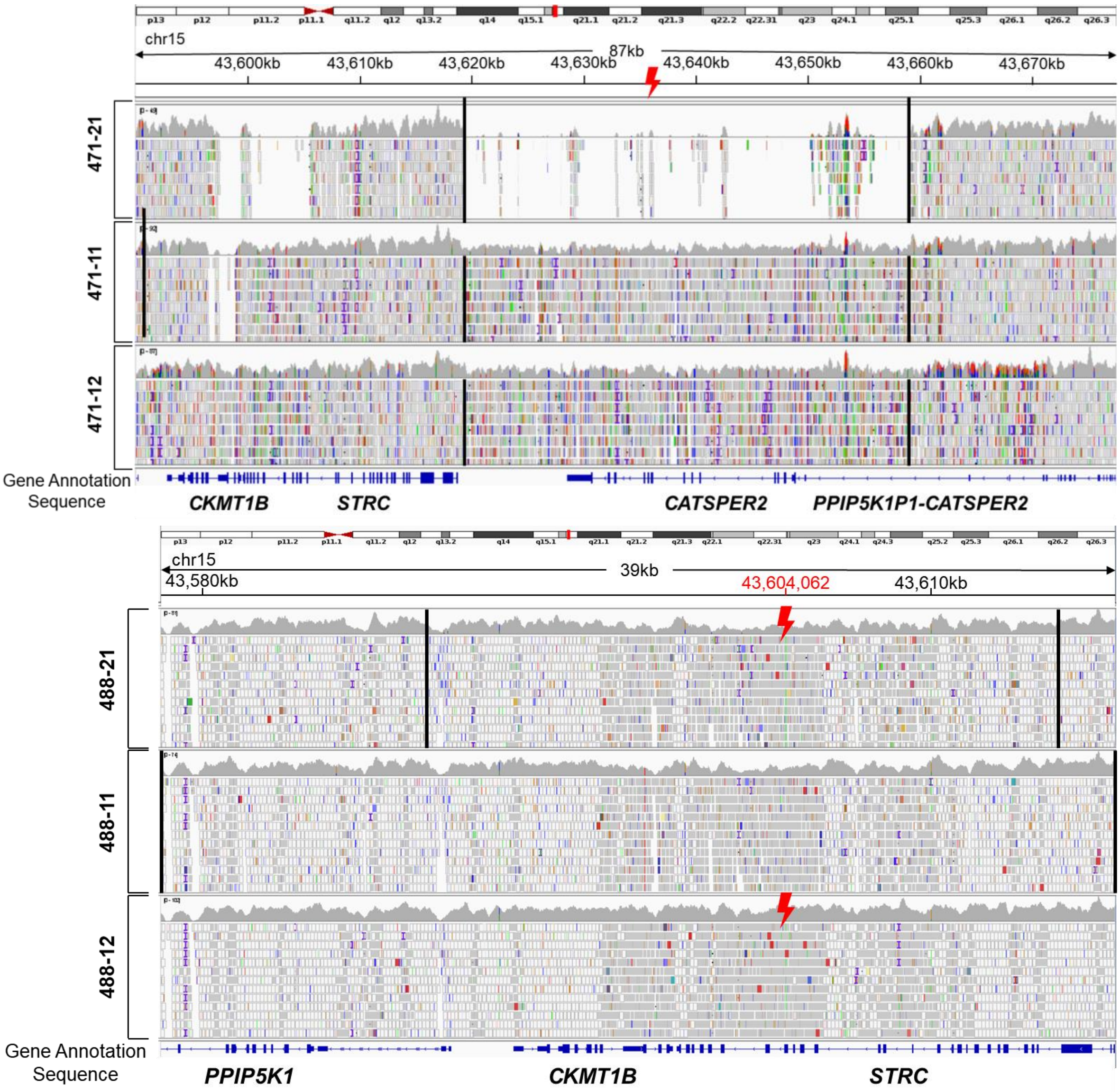

Supplementary Fig. 5. Visualization of detected gross copy number deletions in YUHL 471 and YUHL 488 trios

Integrative Genomics Viewer images of the copy number deletions in *STRC* gene regions of YUHL471 and YUHL488 trios. The sequencing coverage (top) and sequencing reads (bottom) are shown. The called regions are bordered by two black lines, -21; proband, -11; father, -12; mother

Supplementary Table 1. Demographics of patients screened by WES and WGS

Clinical characteristics		WGS	WES
Age of Onset	Pre	57/140 (40.7%)	85/437 (19.4%)
	Post	83/140 (59.3%)	347/437 (79.4%)
	Unknown	0/140 (0%)	5/437 (1.1%)
Mode of Inheritance	AD|XL	47/140 (33.6%)	196/437 (44.9%)
	AR	93/140 (66.4%)	240/437 (54.9%)
	Unknown	0/140 (0%)	1/437 (0.2%)
Simplex/Multiplex	Simplex	58/140 (41.4%)	152/437 (34.8%)
	Multiplex	82/140 (58.6%)	285/437 (65.2%)
Syndromic Feature	SHL	5/140 (3.6%)	16/437 (3.7%)
	NSHL	135/140 (96.4%)	417/437 (95.4%)
	Unknown	0/140 (0%)	4/437 (0.9%)
Severity	Mild	11/140 (7.9%)	47/437 (10.8%)
	Moderate	52/140 (37.1%)	236/437 (54%)
	Severe	22/140 (15.7%)	61/437 (14%)
	Profound	55/140 (39.3%)	91/437 (20.8%)
	Unknown	0/140 (0%)	2/437 (0.5%)
Vertigo	Yes	31/140 (22.1%)	109/437 (24.9%)
	No	109/140 (77.9%)	310/437 (70.9%)
	Unknown	0/140 (0%)	18/437 (4.1%)

Supplementary Table 2. Variants information of diagnosed patients by WGS.

Patient[a]	Gene	hg38	Accession	cDNA	Amino acid	Conservation	dbSNP[b]	Zygosity	gnomAD MAF[c]	gnomAD EAS[d]	gnomAD KOR[e]	Mutation Taster[f]	PP2[g]	SIFT[h]	CADD[i]	DVD[j]	Clinvar[k]	ACMG Classification[l]
YUHL95-21	*MYO15A*	chr17:18119700_T/-	NM_016239.4	c.900delT	p.P301Rfs*143	-	rs2142242418	Het	0.0000006211	0.00002231	-	-	-	-	-	-	-	LP (PVS1,PM2)
		chr17:18119978_-/C		c.1179dupC	p.E396Rfs*35	-	rs772536599	Het	0.00003225	0.0002456	0.001310	-	-	-	-	P	P	LP (PVS1,PM2)
YUHL231-21	*MYO15A*	chr17:18157832_C/T	NM_016239.4	c.C889T	p.R2967*	-	rs944487015	Het	0.000003771	0	0	A (0.81)	-	-	35	VUS	-	LP (PVS1,PM2)
		chr17:18172203_C/G		c.C10263G	p.I342M	*Danio rerio*	rs748246442	Het	0.00003903	0.001381	0.001310	DC (0.488)	Dam (0.621)	Del (0.01)	23.6	LP	-	VUS (PM2_Supporting)
YUHL728-21	*MYO15A*	chr17:18119978_-/C	NM_016239.4	c.1179dupC	p.E396Rfs*35	-	rs772536599	Hom	0.00003225	0.0002456	0.001310	-	-	-	-	P	P	LP (PVS1,PM2)
YUHL 748-21	*MYO15A*	chr17:18172203_C/G	NM_016239.4	c.C10263G	p.I342M	*Danio rerio*	rs748246442	Het	0.00003903	0.001381	0.001310	DC (0.488)	Dam (0.621)	Del (0.01)	23.6	LP	-	VUS (PM2_Supporting)
		chr17:18172292_T/G		c.10350+2T>G	-	-	rs760148486	Het	0.0000006195	0.00002228	0.0002619	-	-	-	24.5	VUS	-	LP (PVS1,PM2)
YUHL 488-21	*STRC*	chr15:43604062_G/A	NM_153700.2	c.C4309T	p.Q1437*	-	rs769246194	Hemi	0.000004959-	0	0	A (1)	-	-	335	-	VUS	LP (PVS1,PM2)
YUHL 85-21	*CDH23*	chr10:71570884_C/T	NM_022124.6	c. C719T	p.P240L	*Danio rerio*	rs121908354	Het	0.00003346	0.001203	0.002357	A (1)	Dam (0.98)	Tol (0.05)	24.6	P	P	VUS (PM2_Supporting)
		chr10:71805818_CGCTCCAA/-		c.7887_7894del	p.S2630Vfs*10	-	-	Het	-	-	-	-	-	-	-	-	-	LP (PVS1,PM2)
YUHL 1118-21	*CDH23*	chr10:71807606_C/T	NM_022124.6	c.C8399T	p.S2800F	*Danio rerio*	-	Het	-	-	-	DC (0.81)	Dam (0.774)	Del (0.477)	25.5	-	-	VUS (PM2)
		chr10:71803233_C/T		c.C7685T	p.S2562L	*Xenopus tropicalis*	rs538435711	Het	0.00002620	0.00004509	0.0002869	DC (0.81)	Ben (0.154)	Del (0.506)	28.3	VUS	VUS	VUS (PM2_Supporting, BP4)

YUHL 844-21	*USH2A*	chr1:215674796-215674799_ATTT/-	NM_206933.4	c.13112_13115del	p.Q4371Rfs*19	-	rs768161313	Het	0.000006196	0.00008915	0.0007899	-	-	-	33	P/LP	P	LP (PVS1,PM2)
		chr1:216086749_G/A		c.C4957T	p.R1653*	-	rs754768875	Het	0.00001178	0.00006687	0	A (1)	-	-	33	P/LP	P	LP (PVS1,PM2)
YUHL 1133-21	*USH2A*	chr1:215877882_T/C	NM_206933.4	c.8559-2A>G	-	-	rs397518039	Het	0.000009916	0.0003345	0.0007891	-	-	-	34	P	P	VUS (PVS1_Moderate,PM2_Supporting)
		chr1:216246592_A/C		c.T2802G	p.C934W	*Danio rerio*	rs201527662	Het	0.00009046	0.002564	0.003684	DC (1)	Dam (0.999)	Del (0)	25.5	P/LP	VUS	VUS (PP3,BS1_Supporting)
YUHL 603-21	*ILDR1*	chr3:122001823_C/G	NM_001199799.2	c.G421C	p.G141R	*Danio rerio*	rs765136820	Het	0.00001735	0.0006237	0.0005238	DC (1)	Dam (0.999)	Del (0)	26.4	-	P	VUS (BS1_Supporting)
		chr3:121725861_G/T		c.C206A	p.P69H	*Danio rerio*	rs754492545	Het	0.000007440	0.0002450	0.0002619	DC (1)	Dam (0.998)	Del (0)	26.8	-	P	VUS (PM2_Supporting,PP3)
YUHL 239-21	*TRIOBP*	chr22:37726245_C/T	NM_001039141.3	c.C3689T	p.P390L	*Mus musculus*	rs200665858	Het	0.00005089	0.001761	0.001572	DC (0.99)	Dam (0.989)	Del (0)	27.6	-	VUS	VUS (PM2_Supporting)
		chr22:37759248_C/T		c.C6308T	p.P2103L	*Mus musculus*	rs200250028	Het	0.00009052	0.001805	0.001572	DC (0.96)	Ben (0.24)	Tol (0.12)	27.6	VUS	-	VUS (PM2_Supporting,BP4)
YUHL 438-21	*TWNK*	chr10:100989382_G/A	NM_021830.5	c.G1172A	p.R391H	*Mus musculus*	rs556445621	Het	0.00002850	0.0002897	0.0005238	DC (0.68)	Dam (0.836)	Del (0.04)	23.4	P/VUS/LB	VUS	VUS (PM1,PM2 _Supporting,PP3)
		chr10:100989406_A/G		c.A1196G	p.N399S	*Danio rerio*	rs863223921	Het	0.000007435	0.00002228	0	DC (0.99)	Dam (0.896)	Tol (0.07)	21.9	P/LP/VUS	P	VUS (PM1,PM2)
YUHL 742-21	*LOXHD1*	chr18:46534334_C/T	NM_144612	c.4212+1G>A	-	-	rs889110926	Het	0.00002389	0.0007086	0.002674	-	-	-	24.2	-	P	VUS (PVS1_Moderate, PM2_Supporting)
		chr18:46579680_G/A		c.C1759T	p.R587W	*Danio rerio*	rs540100675	Het	0.00007153	0.0001222	0	DC (0.93)	Dam (0.999)	Del (0)	33	VUS	LP	VUS (PM2)
YUHL20-21	*CHD7*	chr8:60860946_-/C	NM_017780	c.7652dupC	p.P2553Tfs*22	-	-	Het	-	-	-	-	-	-	33	P	P	LP (PVS1,PM2)

YUHL 67-21	*CHD7*	chr8:60742366_C/T	NM_017780	c.C934T	p.R312*	-	-	Het	-	-	-	A (1)	-	-	37	P	P	LP (PVS1,PM2)
YUHL 199-21	*CHD7*	chr8:60853179_C/T	NM_017780	c.C6454T	p.Q2152*	-	-	Het	-	-	-	A (1)	-	-	40	P	P	LP (PVS1,PM2)
YUHL 477-21	*TECTA*	chr11:121109260_C/T	NM_005422	c.C248T	p.T83M	*Danio rerio*	rs145898158	Het	0.00009789	0.0001114	0.0002621	DC (1)	Dam (0.899)	Del (0.518)	27.1	P/VUS/LB	LP	VUS (PM2)
YUHL 749-21	*TECTA*	chr11:121113638_C/T	NM_005422	c.C710T	p.T237I	*Danio rerio*	-	Het	-	-	-	DC (0.99)	Dam (0.999)	Del (0)	25.8	-	P	VUS (PM2, PP3)
YUHL 1113-21	*KCNQ4*	chr1:40784233_T/C	NM_004700	c.T140C	p.L47P	*Danio rerio*	rs1271250198	Het	0.00002544	0.001156	-	DC (0.81)	Ben (0.093)	Tol (0.365)	16.52	P	P	VUS (PM2)
YUHL 1119-21	*KCNQ4*	chr1:40784233_T/C	NM_004700	c.T140C	p.L47P	*Danio rerio*	rs1271250198	Het	0.00002544	0.001156	-	DC (0.81)	Ben (0.093)	Tol (0.365)	16.52	P	P	VUS (PM2)
YUHL 464-21	*POU4F3*	chr5:146339986_C/G	NM_002700.3	c.C559G	p.L187V	*Danio rerio*	rs1200909411	Het	0.000001860	0.00006685	0	DC (1)	Dam (0.999)	Del (0)	26.5	-	VUS	VUS (PM2, PP3)
YUHL 1136-21	*POU4F3*	chr5:146340390_G/C	NM_002700.3	c.G963C	p.W321C	*Danio rerio*	-	Het	-	-	-	DC (1)	Dam (1)	Del (0)	32	-	-	VUS (PM2, PP3)
YUHL 1143-21	*WFS1*	chr4:6301752_C/T	NM_006005.3	c.C1957T	p.R653C	*Xenopus tropicalis*	rs201064551	Het	0.0002832	0.0008468	0.001048	DC (1)	Dam (0.953)	Del (0.007)	33	LP/VUS/LB	LP	VUS (PP3, BS1)
YUHL 187-21	*GRHL2*	chr8:102643941_A/G	NM_024915.4	c.A1334G	p.Q445R	*Xenopus tropicalis*	rs145717789	Het	0.00003224	0.001137	0.0005311	N (0.7)	Ben (0)	Tol (0.62)	17.64	-	LP	VUS (BP4)
YUHL 975-21	*MYO7A*	chr11:77156018_C/G	NM_000260.4	c.C397G	p.H133D	*Danio rerio*	rs111033403	Het	0.0000006196	0	0	DC (1)	Dam (0.998)	Del (0.04)	27.3	VUS	P	VUS (PM2, PM5, PP3)
YUHL 1107-21	*MYH9*	chr22:36285744_G/A	NM_002473.6	c.C5188T	p.R1730C	*Danio rerio*	rs201021615	Het	0.00007197	0.001205	0.0005297	DC (1)	Dam (0.952)	Del (0)	32	VUS	P	VUS (PP3, BS1)

YUHL 1153-21	*MYH14*	chr19:50292309_C/T	NM_001145809.2	c.C5176T	p.R1726W	*Danio rerio*	rs372062358	Het	0.00002252	0.0002036	0.0005631	DC (0.96)	Dam (0.989)	Del (0)	33	VUS	P	VUS (-)
YUHL 301-21	*DIAPH1*	chr5:141576797_A/T	NM_005219.5	c.T1355A	p.F452Y	*Xenopus tropicalis*	rs757579900	Het	0.0000006196	0.00002228	0.0002619	DC (0.99)	Dam (0.855)	Del (0)	28.1	-	VUS	VUS (PM2, PP3)
YUHL 174-21	*LMX1A*	chr1:165249528_C/T	NM_001174069.2	c.G376A	p.E126K	*Gallus gallus*	rs376042184	Het	0.000004956	0.0001114	0.0002619	DC (0.99)	Dam (0.713)	Tol (0.13)	24.9	-	VUS	VUS (PM2)
YUHL 609-21	*SEMA3E*	chr7:83490113_C/T	NM_001178129.2	c.96+1G>A	-	-	-	Het	-	-	-	-	-	-	33	-	-	VUS (PM2)

A, automated; AD, autosomal dominant; AR, autosomal recessive; Ben, benign; Dam, probably damaging; Del, deleterious; DC, disease-causing; Het, heterozygous in the affected individual; Hom, homozygous in the affected individual; Hemi, hemizygous in the affected individual; LP, likely pathogenic; P, pathogenic; Tol, tolerated; VUS, variant of unknown significance;

[a]Individual indicate affected patients in whom variants were found, comprising one case cohort.

[b]dbSNP database (http://www.ncbi.nlm.nih.gov/SNP).

[c,d,e]Population frequency of variants in gnomAD, EAS(East Asian) and KOR(Korean) database (https://gnomad.broadinstitute.org/).

[f]MutationTaster (http://www.mutationtaster.org/).

[g]PolyPhen-2 HumVar prediction score (http://genetics.bwh.harvard.edu/pph2/).

[h]SIFT Sorting Intolerant from Tolerant (http://sift.jcvi.org/).

[i]phred-like scores (scaled C-scores) on the Combined Annotation-Dependent Depletion (http://cadd.gs.washington.edu/home/).

[j]DVD, Deafness variation database. (https://deafnessvariationdatabase.org/)

[k]Clinvar, public archive with free access to reports on the relationships between human variations and phenotypes, with supporting evidence. (https://www.ncbi.nlm.nih.gov/clinvar/).

[l]Classifications of variants are based on VIP-HL (http://hearing.genetics.bgi.com/), an online platform for classifying variants according to the ACMG guidelines adapted for hearing loss.

Supplementary Table 3. Splice-altering variant candidates detected in WGS.

Patient[a]	Gene	Position (hg38)	cDNA position	SpliceAI Delta Score[b]				SpliceAI Delta Position[c]			
				Accept or Gain	Accept or Loss	Donor Gain	Donor Loss	Accept or Gain	Accept or Loss	Donor Gain	Donor Loss
YUHL121-21	*EYA1*	chr8:71299239_A/T	c.640-6T>A	0.98	0.63	0	0	-2	-6	-1	-36
YUHL704-21	*CDH23*	chr10:71791123_G/A	c.6050-9G>A	0.99	0.83	0	0	2	9	8	24
		chr10:71790959_G/A	c.6050-173G>A	0	0	0.63	0	-3	13	1	13
YUHL34-21	*TECTA*	chr11:121137419_A/G	c.2942-2A>G	0.16	0.88	0	0	10	2	17	2
YUHL183-21	*LMX1A*	chr1:165353091_T/C	c.248A>G	0	0	0.91	0.1	-1	13	0	-15
YUHL209-21	*MYO15A*	chr17:18136900_G/C	c.4779+214G>C	0.15	0	0	0	-34	0	-36	0
YUHL615-21	*OTOA*	chr16:21700868_C/A	c.841-20C>A	0.28	0.11	0	0	2	20	20	28
YUHL1049-21	*OTOG*	chr11:17556178_A/G	c.695+281A>G	0	0	0.32	0	-46	-22	-1	41
		chr11:17593310_G/T	c.3160G>T	0	0	0	0.28	17	-46	-2	17
YUHL1123-21	*CDH23*	chr10:71702283_G/A	c.2587+72G>A	0	0	0.26	0	-31	49	1	-16
		chr10:71489118_G/A	c.146-20964G>A	0.21	0	0	0	2	4	2	-38
YUHL1156-21	*MYO7A*	chr11:77213610_G/T	c.4779+214G>C	0.12	0	0.02	0	10	1	9	-5
YUHL1157-21	*OTOF*	chr2:26486603_G/A	c.1046-1970C>T	0.23	0	0.1	0	-14	45	-45	45
		chr2:26465009_C/T	c.4820G>A	0.59	0	0	0	-2	20	-2	-47

[a]Individual indicate affected patients in whom variants were found, comprising one case cohort.
[b] Delta score of a variant, defined as the maximum of (DS_AG, DS_AL, DS_DG, DS_DL), ranges from 0 to 1 and can be interpreted as the probability of the variant being splice-altering.
[c] Delta position conveys information about the location where splicing changes relative to the variant position (positive values are downstream of the variant, negative values are upstream).

Supplementary Table 4. Mitochondrial variant candidates detected in WGS.

Patient[a]	Gene	Position	DVD[b]	gnomAD[c]		Mitomap[d]				
				homoplasmic AF	heteroplasmic AF	Count	Coverage	status	GBCNT	GBFREQ
YUHL390-21	*MT-RNR1*	chrM:1555_A/G	P	0.001117	0.000195	4572	4572	Cfrm	80	0.14
YUHL681-21						2022	2022			
YUHL847-21						1346	1347			
YUHL58-21	*MT-CO1*	chrM:7444_G/A	B	0.005353	0.0001772	2714	2716	Reported	194	0.34
YUHL1112-21	*MT-ND1*	chrM:3396_T/C	LB	0.01508	0.0001241	2363	2363	Reported	420	0.74
YUHL972-21	*MT-RNR1*	chrM:1310_C/T	LB	0.0003367	0	8657	8660	Reported	38	0.07
YUHL560-21						2439	2459			
YUHL1137-21	*MT-TW*	chrM:5558_A/G	-	0.002375	0.0007089	3126	3127	Reported	106	0.19
YUHL1137-21	*MT-TC*	chrM:5821_G/A	-	0.002588	0.0001063	2894	2898	Reported	339	0.60
YUHL1148-21	*MT-TR*	chrM:10454_T/C	-	0.003987	0.00001772	1853	1853	Reported	190	0.33
YUHL1168-21						2236	2236			
YUHL1173-21	*MT-ND5*	chrM:13528_A/G	-	0.002091	0.00005317	2478	2481	Reported	72	0.18
YUHL188-21	*MT-TE1*	chrM:14693_A/G	-	0.001932	0.00007089	740	742	Reported	329	0.58
YUHL566-21						4563	4564			
YUHL681-21						1776	1776			
YUHL1174-21	*MT-TT*	chrM:15901_A/G	-	0.00005316	0	1667	1667	Reported	9	0.02

GBCNT; Count of allele count of control data from GenBank; GBFREQ; GBFREQ; allele frequency from GenBank
[a]Individual indicate affected patients in whom variants were found, comprising one case cohort.
[b]DVD, Deafness variation database. (https://deafnessvariationdatabase.org/)
[c]Population frequency of variants in gnomAD database (https://gnomad.broadinstitute.org/).
[d]Mitomap, a human mitochondrial genome database (https://www.mitomap.org/MITOMAP).

Supplementary Table 5. Clinical characteristics of patients harboring the m.1555A>G & m.7444G>A mutation

Patient[a]	Inheritance	Onset	Sideness	Severity	Progressiveness	PTA(dBHL)		Pattern	Exposure to Aminoglycoside	Additional history
						Right	Left			
YUHL390-21	Familial	Congenital	both	profound	stable	107.5	120	flat	No	-
YUHL681-21	Familial	Mid 20s	both	moderate	progressive	46.25	46.25	ski-sloping	No	Hearing loss of mother initiated after antibiotics administration
YUHL847-21	Familial	Late 40s	both	profound (Rt) / mild (Lt)	progressive	118.75	25	flat	No	
YUHL58-21	Familial	Early 0s	both	moderate	progressive	47.5	48.75	flat	No	-

PTA; Pure Tone Audiometry
[a]Individual indicate affected patients in whom variants were found, comprising one case cohort.

Supplementary Table 6. Copy number variant candidates detected in WGS.

Patient[a]	Gene	Chromosome	Start	End	SV length	SV type	Read Depth	GT:CN	Location	gnomAD AF[b]	AnnotSV ranking[c]
YUHL471-21	*STRC*	15	43564001	43697200	-133200	DEL	0.319508	0/1:1	txStart-txEnd	0.0001381	5
YUHL488-21	*STRC*	15	43589201	43615200	-26000	DEL	0.653585	0/1:1	intron4-txEnd	-	5
YUHL488-11	*STRC*	15	43578301	43617600	-39300	DEL	0.676284	0/1:1	exon2-txEnd	-	5
YUHL58-21	*STRC*	15	43562601	43653400	-90800	DEL	0.610974	0/1:1	txStart-txEnd	0.0001381	5
YUHL284-21	*COL1A1*	17	50184401	50185600	-1200	DEL	0.335734	0/1:1	exon51-exon51	-	4
YUHL827-21	*CHD7*	8	60678601	60679800	-1200	DEL	0.124248	1/1:0	txStart-intron1	-	4
YUHL843-21	*PTPRQ*	12	80601751	80620050	-18300	DEL	0.612192	0/1:1	intron26-intron31	0.00001586	4
YUHL972-21	*CHD7*	8	60678451	60680700	-2250	DEL	0.258161	1/1:0	txStart-intron1	0.0003103	4
YUHL975-21	*SLC12A2*	5	128083351	128084400	-1050	DEL	0.372807	0/1:1	txStart-exon1	-	4
YUHL1131-21	*ABHD12*	20	25384201	25428150	-43950	DEL	0.503705	0/1:1	txStart-intron1	0.000007931	4
YUHL1113-21	*PTPRQ*	12	80464201	80479200	-15000	DEL	0.500758	0/1:1	intron6-intron8	0.000007931	3

CN, copy number; GT; zygosity, 0/1 indicates heterozygous variant and 1/1 indicates homozygous variant; SV, structural variant
[a]Individual indicate affected patients in whom variants were found, comprising one case cohort.
[b]Population frequency of variants in gnomAD (https://gnomad.broadinstitute.org/).
[c]A ranking score to assess SV pathogenicity by the joint consensus recommendation of ACMG and ClinGen (Riggs et al., 2020). Data are available from (https://lbgi.fr/AnnotSV/)

Supplementary Table 7. Transposable element insertion candidates detected in WGS.

Patient[a]	Gene	Position (hg38)	TSD	Type	Subtype	GT	AF_DISC	LCOV	RCOV
YUHL1109-21	*SLC17A8*	chr12:100371388	AAAAAAAAGTCTCTG	Alu	two_side_tprt_both	0/1	37	29.285	36.31
YUHL471-21<br>YUHL 1107-21	*CDH23*	chr10:71433771	CCCTCTCAGCTCTACTTTG	Alu	two_side_tprt_both	1/1	30<br>59	16.04<br>48.7	33.3<br>35.96
YUHL847-21	*EYA1*	chr8:71561825	CTGAAAGTTTT	Alu	two_side_tprt_both	0/1	16	18.385	23.435
YUHL239-21	*PAX3*	chr2:222255919	TTTTTTTTTTTTTTTTTTTTTGT	Alu	two_side_tprt_both	0/1	16	31.805	26.895
YUHL1112-21	*PAX3*	chr2:222226161	AGAAAGAGAATTCGC	Alu	two_side_tprt_both	0/1	28	47.095	35.925
YUHL1148-21	*TMC1*	chr9:72584784	TTTTTTTTTTTTTTTTTT	Alu	two_side_tprt_both	0/1	21	34.52	31.575

AF_DISC, Number of effective discordant pairs (for estimated allele frequency); GT, zygosity and 0/1 indicates heterozygous variant; LCOV, Left focal coverage; RCOV, Right focal coverage; TSD, target site duplication; Type, type of structural variant; two_side_tprt_both, two-sided target-primed reverse transcription (TPRT);

[a]Individual indicate affected patients in whom variants were found, comprising one case cohort.

Supplementary Table 8. Cis-regulatory variant candidates detected in WGS.

Patient[a]	Gene	Position (hg38)	cDNA position	dbSNP[b]	gnomAD MAF[c]	CADD[d]	Clinvar[e]	DVD[f]	Regulatory Feature[g]
YUHL26-21	*CDH23*	chr10:71548093_C/G	c.430-18649C>G	rs1184115420	0.00001314	17.4	-	-	Distal-enhancer like signature, CTCF-bound
YUHL518-21	*OTOF*	chr2:26491893_A/G	c.898-2153T>C	-	-	4.469	-	-	Distal-enhancer like signature
YUHL470-21	*MYO6*	chr6:75842807_T/C	c.816+1429T>C	-	-	14.2	-	-	Distal-enhancer like signature
YUHL1123-21	*WHRN*	chr9:114473312_C/T	c.837+5241G>A	rs572787776	0.0001445	0.533	-	VUS	Distal-enhancer like signature, CTCF-bound
YUHL1148-21	*COL4A4*	chr2:227065324_G/C	c.1988-2726C>G	rs1444994654	0.00002627	2.03	-	VUS	Distal-enhancer like signature, CTCF-bound
YUHL1156-21	*CDH23*	chr10:71621341_T/C	c.1134+3948T>C	-	-	0.074	-	-	Distal-enhancer like signature, CTCF-bound

VUS, variant of unknown significance
[a]Individual indicate affected patients in whom variants were found, comprising one case cohort.
[b]dbSNP database (http://www.ncbi.nlm.nih.gov/SNP).
[c]Population frequency of variants in gnomAD database (https://gnomad.broadinstitute.org/).
[d]phred-like scores (scaled C-scores) on the Combined Annotation-Dependent Depletion (http://cadd.gs.washington.edu/home/).
[e]Clinvar, public archive with free access to reports on the relationships between human variations and phenotypes, with supporting evidence (https://www.ncbi.nlm.nih.gov/clinvar/).
[f]DVD, Deafness variation database. (https://deafnessvariationdatabase.org/)
[g]Regulatory feature of cis-regulatory element retrieved from SCREEN (Search Candidate cis-Regulatory Elements by ENCODE) database (https://screen.encodeproject.org/)

Supplementary Table 9. Variant information of patients screened for CMV infection using WGS.

Patient[a]	Gene	hg38	Accession	cDNA	Amino acid	Conservation	dbSNP[b]	Zygosity	gnomAD MAF[c]	gnomAD EAS[d]	gnomAD KOR[e]	Mutation Taster[f]	PP2[g]	SIFT[h]	CADD[i]	DVD[j]	Clinvar[k]	CMV reads	Aligned portion (%)
YUHL 742-21	*LOXHD1*	chr18:46534334_C/T	NM_144612	c.4212+1G>A	-	-	rs889110926	Het	0.00002389	0.0007086	0.002674	-	-	-	33	P	-	110,046	0.15% (110,046/71,524580)
		chr18:46579680_G/A		c.C1759T	p.R587W	*Danio rerio*	rs540100675	Het	0.00007153	0.0001222	0	D(0.933513)	D(0)	-	29.8	LP	VUS		
YUHL 1180-21	*KCNQ4*	chr1:40784233_C/T	NM_004700	c.T140C	p.L47P	*Danio rerio*	rs1271250198	Het	0.00002544	0.001156	-	D(1)	B(0.049)	D(0.02)	24.2	P	P	1,610	0.08% (1,610/1,988,346)

AD, autosomal dominant; AR, autosomal recessive; B, benign; D, probably damaging; DC, disease-causing; Del, deleterious; Het, heterozygous in the affected individual; LP, likely pathogenic; P, pathogenic; VUS, variant of unknwon significance

[a]Individual indicate affected patients in whom variants were found, comprising one case cohort.

[b]dbSNP database (http://www.ncbi.nlm.nih.gov/SNP).

[c,d,e]Population frequency of variants in gnomAD, EAS(East Asian) and KOR(Korean) database (https://gnomad.broadinstitute.org/).

[f]MutationTaster (http://www.mutationtaster.org/).

[g]PolyPhen-2 HumVar prediction score (http://genetics.bwh.harvard.edu/pph2/).

[h]SIFT Sorting Intolerant from Tolerant (http://sift.jcvi.org/).

[i]phred-like scores (scaled C-scores) on the Combined Annotation-Dependent Depletion (http://cadd.gs.washington.edu/home/).

[j]DVD, Deafness variation database. (https://deafnessvariationdatabase.org/)

[k]Clinvar, public archive with free access to reports on the relationships between human variations and phenotypes, with supporting evidence. (https://www.ncbi.nlm.nih.gov/clinvar/).

Supplementary Table 10. Pathogenic variants detected in ACMG SF genes in WGS.

Patient[a]	Gene	hg38	Accession	cDNA	Amino acid	Conservation	dbSNP[b]	Zygosity	gnomAD MAF[c]	gnomAD EAS[d]	gnomAD KOR[e]	Mutation Taster[f]	PP2[g]	SIFT[h]	CADD[i]	DVD[j]	Clinvar[k]	ACMG SF v3.2[l]
YUHL 1049-21,YUHL 162-21	*MUTYH*	chr1:45332446_G/A	NM_001350650	c.C304T	p.R102C	*Danio rerio*	rs200495564	Het	2.79E-05	0	0.000262	DC (0.81)	Dam (0.875)	Del (0.912)	29.5	-	P	MUTYH-associated polyposis (AD)
YUHL 150-21		chr1:45332458_G/A	NM_001350650	c.C292T	p.R98W	*Danio rerio*	rs34126013	Het	2.09E-05	0.0003	0	DC (0.81)	Dam (0.971)	Del (0.912)	33	-	P/LP	
YUHL 1049-21, YUHL 1095-21, YUHL 1105-21, YUHL 1118-21, YUHL 1147-21, YUHL 1151-21, YUHL 1156-21, YUHL 1168-21,	*MSH2*	chr2:47414420_T/-	NM_001258281	c.744+2T>-	-	-	rs587779194	Het	0.0007	0.0023	-	-	-	-	-	-	LP	Lynch syndrome (AD)
YUHL 827-21	*TTN*	chr2:178530861_G/A	NM_003319	c.C78559T	p.R26187*	*Danio rerio*	-	Het	1.4E-05	0	-	A (0.81)	-	-	69	-	P/LP	Dilated cardiomyopathy (truncating variants only) AD
YUHL 1153-21	*DES*	chr2:219420150_C/T	NM_001382708	c.C634T	p.R212*	*Danio rerio*	rs781590560	Het	6.98E-06	0	0	A (0.81)	-	-	38	-	P	Dilated myopathy (AD) Myofibrillar myopathy (AD)
YUHL 827-21	*BTD*	chr3:15644487_C/T	NM_001281723	c.C571T	p.R191C	*Gallus gallus*	rs372844636	Het	1.4E-05	0	0.000262	DC (0.81)	Dam (0.782)	Del (0.784)	34	P	P	Biotinidase deficiency (AR)
YUHL 464-21	*BRCA2*	chr13:32332877_A/T	NM_000059	c.A1399T	p.K467*	*Mus musculus*	rs80358427	Het	-	-	0.000262	A (0.81)	-	-	35	-	P	Hereditary breast and ovarian cancer (AD)
YUHL 1127-21		chr13:32336936_-/A	NM_000059	c.2582dupA	p.N863Kfs*18	-	rs606231399	Het	6.99E-06	0	-	-	-	-	-	-	P	
YUHL 560-21		chr13:32356472_C/T	NM_000059	c.C7480T	p.R2494*	*Danio rerio*	rs80358972	Het	1.4E-05	0	0.000524	A (0.81)	-	-	50	-	P	
YUHL 847-21	*ATP7B*	chr13:51950334_T/-	NM_001005918	c.2027delA	p.K676Sfs*35	*Danio rerio*	rs777362050	Het	-	-	0.000524	-	-	-	-	-	LP	Wilson disease (AR)
YUHL 1107-21	*TP53*	chr17:7674972_C/T	NM_001126114	c.560-1G>A	-	-	-	Het	6.98E-06	0.0003	-	DC (0.81)	-	-	23.6	-	P	Li-Fraumeni

																		syndrome (AD)
YUHL 277-21	*GAA*	chr17:80108818_T/A	NM_000152	c.T1316A	p.M439K	*Mus musculus*	rs747610090	Het	-	-	0.001769	DC (0.394)	Ben (0.342)	Del (0.721)	22.9	-	P/LP	Pompe disease (AR)
YUHL 33-21	*LDLR*	chr19:11116209_C/G	NM_001195800	c.C1198G	p.L400V	*Danio rerio*	-	Het	-	-	-	DC (0.588)	Dam (0.719)	Del (0.784)	24.1	-	P/LP	Familial hypercholesterolemia (AD)

A, automated; AD, autosomal dominant; AR, autosomal recessive; Ben, benign; Dam, probably damaging; Del, deleterious; DC, disease-causing; Het, heterozygous in the affected individual; LP, likely pathogenic; P, pathogenic

[a]Individual indicate affected patients in whom variants were found, comprising one case cohort.

[b]dbSNP database (http://www.ncbi.nlm.nih.gov/SNP).

[c,d,e]Population frequency of variants in gnomAD, EAS(East Asian) and KOR(Korean) database (https://gnomad.broadinstitute.org/).

[f]MutationTaster (http://www.mutationtaster.org/).

[g]PolyPhen-2 HumVar prediction score (http://genetics.bwh.harvard.edu/pph2/).

[h]SIFT Sorting Intolerant from Tolerant (http://sift.jcvi.org/).

[i]phred-like scores (scaled C-scores) on the Combined Annotation-Dependent Depletion (http://cadd.gs.washington.edu/home/).

[j]DVD, Deafness variation database. (https://deafnessvariationdatabase.org/)

[k]Clinvar, public archive with free access to reports on the relationships between human variations and phenotypes, with supporting evidence. (https://www.ncbi.nlm.nih.gov/clinvar/).

[l]ACMG Recommendations for Reporting of Secondary Findings in Clinical Exome and Genome Sequencing (https://www.ncbi.nlm.nih.gov/clinvar/docs/acmg/)
